# Efficacy and safety of prostaglandin drugs for elevated intraocular pressure: a Bayesian network meta-analysis

**DOI:** 10.3389/fmed.2025.1642986

**Published:** 2025-08-11

**Authors:** Jieting Peng, Wenrui Huang, Junguo Duan

**Affiliations:** ^1^Chengdu University of Traditional Chinese Medicine, Affiliated Eye Hospital of Chengdu University of Traditional Chinese Medicine, Chengdu, China; ^2^Guangzhou University of Chinese Medicine, Shenzhen Traditional Chinese Medicine Hospital, Guangzhou, China; ^3^Chengdu University of Traditional Chinese Medicine, Yinhai Eye Hospital Affiliated to Chengdu University of Traditional Chinese Medicine, Chengdu, China

**Keywords:** intraocular pressure, glaucoma, prostaglandin analogs, network meta-analysis, Bayesian

## Abstract

**Objective:**

To evaluate and compare the effectiveness and safety of latanoprost, bimatoprost, travoprost, and tafluprost in lowering intraocular pressure (IOP) in individuals with glaucoma or ocular hypertension.

**Methods:**

We searched PubMed, Embase, Web of Science, and the Cochrane Library for randomized controlled trials (RCTs) published up to April 2025 comparing latanoprost, bimatoprost, travoprost, and tafluprost in adults with glaucoma or ocular hypertension. Primary outcomes were IOP reduction and conjunctival hyperemia. We assessed study quality using the Cochrane Risk of Bias 2.0 tool. Evidence certainty was evaluated with the CINeMA framework. A Bayesian network meta-analysis was conducted in RStudio. This review is registered with PROSPERO (CRD420251034803).

**Results:**

25 RCTs published between 2001 and 2024, involving 4,045 participants, were included. All studies compared monotherapy with latanoprost, bimatoprost, travoprost, or tafluprost. Among these, bimatoprost showed the most effective reduction in intraocular pressure compared to latanoprost [mean difference (MD) 0.69; 95%confidence interval (CI) 0.28–1.1; SUCRA 95.6%; moderate confidence]. It also performed significantly better than travoprost (MD 0.64; 0.14–1.09; 39.2%; low confidence). No other comparisons showed statistically significant differences. Overall, the quality of evidence for this outcome ranged from low to moderate. In terms of safety, 16 trials, including 3,119 participants, reported on conjunctival hyperemia. Both bimatoprost [odds ratio (OR) 3.3; 2.5–4.5; 18.4%, high confidence] and travoprost (0.46; 0.33–0.63; 55%, high confidence) were associated with a higher risk of hyperemia compared to latanoprost. Bimatoprost also posed a significantly greater risk than travoprost (1.51; 1.06–2.16, high confidence).

**Conclusion:**

Bimatoprost provided the greatest IOP reduction but carried a higher risk of conjunctival hyperemia. Latanoprost and tafluprost offered balanced efficacy with better tolerability, making them suitable for patients with mild disease.

**Systematic review registration:**

https://www.crd.york.ac.uk/PROSPERO/view/CRD420251034803.

## Introduction

Elevated intraocular pressure (IOP) is a key factor in ocular health and the most significant modifiable risk factor for glaucoma and optic nerve damage (1). Glaucoma is a progressive condition that can lead to permanent vision loss if untreated, and elevated IOP is a primary driver of its progression (2). Effective IOP management is, therefore, crucial to slowing disease progression and protecting vision (3).

Medication, laser treatments, and surgeries are therapies for elevated IOP (4). Prostaglandin analogs are the first choice for pharmacological treatment due to their high efficacy and convenient dosing schedule. These drugs lower IOP by increasing uveoscleral outflow, a vital pathway for aqueous humor drainage (5). Common agents include latanoprost (LAT), bimatoprost (BIM), travoprost (TRA), and tafluprost (TAF). These agents are valued for their reliable results and minimal systemic side effects, making them essential in managing elevated IOP (6).

Although prostaglandin drugs are widely used in clinical practice, comprehensive comparisons of all available options are limited. Most studies focus on pairwise comparisons or use inconsistent methods to evaluate efficacy and safety (7–10). This fragmented approach has resulted in conflicting findings, creating uncertainty about these drugs’ relative benefits and risks. A Bayesian network meta-analysis (NMA) offers a powerful solution to these gaps. Combining data from multiple studies enables indirect comparisons of drugs that have not been directly compared in trials (11). This study aims to use Bayesian NMA to evaluate and compare the efficacy and safety of prostaglandin drugs for reducing IOP. This study used Bayesian NMA to evaluate and compare the efficacy and safety of prostaglandin drugs, aiming to refine treatment guidelines for glaucoma and elevated IOP.

## Methods

This NMA was carried out in accordance with the PRISMA Extension guidelines, explicitly designed for systematic reviews involving network meta-analyses of healthcare interventions (12). A comprehensive PRISMA checklist can be found in Appendix 1. The review protocol has also been officially registered with the International Prospective Register of Systematic Reviews (PROSPERO: CRD420251034803).

### Inclusion criteria

#### Participants

The study population comprised patients diagnosed with glaucoma or ocular hypertension, aged 18 years or older. There were no restrictions based on gender, race, or ethnicity.

#### Interventions

The control group consisted of patients receiving LAT monotherapy. The experimental groups included those treated with BIM monotherapy, TRA monotherapy, or TAF monotherapy. Only relevant groups were selected for analysis in clinical trials involving multiple treatment arms to maintain coherence and consistency.

#### Outcome measures

The primary efficacy outcome was intraocular pressure reduction (IOPR), defined as the difference between baseline IOP and endpoint IOP, with a study duration of 3 months. The IOPR and its standard deviation (SDIOPR) were calculated as follows: IOPR = IOP_baseline_ - IOP_endpoint_; SDIOPR = √(SD_baseline_^2^ + SD_endpoint_^2^-2*r*SD_baseline_*SD_endpoint_) (13). Since none of the included studies reported the standard deviation of change, the correlation coefficient (r) was assumed rather than derived. We set r = 0.5, reflecting a moderate level of measurement repeatability commonly accepted in previous literature. This assumption was made to balance the potential variability between baseline and endpoint measurements and to support the robustness and reliability of the results (13). The safety outcome was the incidence of conjunctival hyperemia.

#### Study design

Only randomized controlled trials (RCTs) were included to ensure robust evidence quality.

### Exclusion criteria

Studies were excluded if they used non-RCT study designs, involved combination therapy with non-prostaglandin IOP-lowering agents, lacked primary outcome measures, or presented insufficient or non-extractable data.

### Search strategy

The databases PubMed, Embase, Web of science and Cochrane Library were systematically searched using a range of terms, including “latanoprost,” “PhXA34,” “PHXA41,” “Xalatan,” “bimatoprost,” “Latisse,” “Lumigan,” “AGN192024,” “AGNA,” “travoprost,” “Travatan Z,” “Travatan,” “tafluprost,” “AFP-168,” “glaucoma,” “ocular hypertension,” “randomized controlled trial,” and “RCT.” To ensure thoroughness, the references of all included studies were also manually reviewed. The search encompassed all relevant literature available from the inception of each database through April 2025. Detailed search strategies are available in Appendix 2.

### Study selection and data extraction

The study selection process involved a structured approach, including initial screening, re-screening, and detailed evaluation. Two independent researchers systematically reviewed the literature using EndNote, employing automated and manual methods to remove duplicates. Studies were assessed based on predefined inclusion and exclusion criteria, and those not meeting the criteria were excluded. Full-text reviews were conducted to eliminate studies lacking relevant outcomes or not adhering to the specified interventions. Discrepancies during the review process were resolved through discussion or, if necessary, consultation with a third reviewer.

Data extraction was performed using a standardized form, organizing data into the following categories: ①general study information (authors, publication year, sample size, participant characteristics); ②methodological details (randomization, allocation concealment, blinding, and completeness of outcome data); ③intervention specifics (details of prostaglandin treatments in experimental and control groups, duration of therapy); and ④outcome measures (IOPR and incidence of conjunctival hyperemia).

### Risk of bias and quality assessment

The risk of bias in the included studies was evaluated using the Cochrane Risk of Bias tool (RoB 2.0) (14). This method assesses five critical domains: issues with the randomization process, deviations from the intended interventions, missing outcome data, measurement of outcomes, and selection of reported results. Two independent reviewers conducted the assessment, resolving any disagreements through discussion or consultation with a third reviewer when necessary. To evaluate the strength of evidence from the network meta-analysis, the Confidence in Network Meta-Analysis (CINeMA) framework was employed. CINeMA assesses evidence quality across six domains: within-study bias, indirectness, imprecision, heterogeneity, inconsistency, and reporting bias. Each domain was carefully examined to provide a comprehensive appraisal of the evidence (15, 16). This rigorous process ensured a nuanced understanding of the certainty of evidence supporting the findings.

### Data synthesis and analysis

The NMA was conducted using Stata SE 17 to generate the network plot, illustrating the relationships among treatments. Bayesian network analysis was performed in RStudio, utilizing the Gemtc and Ggplot2 packages. The analysis employed Monte Carlo Markov Chain (MCMC) methods within a consistency model (17). Four MCMC chains were run with 30,000 burn-in iterations and 50,000 sampling iterations. Convergence was assessed through trace plots of posterior sample values over iterations, with overlapping and stable plots indicating successful convergence. A league table was then constructed to present the results quantitatively. For dichotomous outcomes, results were expressed as odds ratios (OR) with 95% confidence intervals (CI), highlighting the relative likelihood of an event occurring between groups. Continuous outcomes were presented as mean differences (MD) with 95% CI, representing the average difference between groups. Heterogeneity was assessed using τ^2^ values, which were classified into four levels: low (<0.04), low-to-moderate (0.04–0.16), moderate-to-high (0.16–0.36), and high (>0.36), following established guidelines (18–20). The ranking of treatments was visualized using cumulative ranking curves and Surface Under the Cumulative Ranking (SUCRA) graphs, created in RStudio. SUCRA values ranged from 0 (indicating a minimal effect) to 1 (indicating a robust effect), providing a clear visualization of the comparative performance of each treatment (21).

To evaluate small-study effects, funnel plot symmetry was assessed in Stata based on direct comparisons. Publication bias was examined for each pairwise comparison individually using estimates from direct evidence.

## Results

### Literature search results

**Figure 1:**
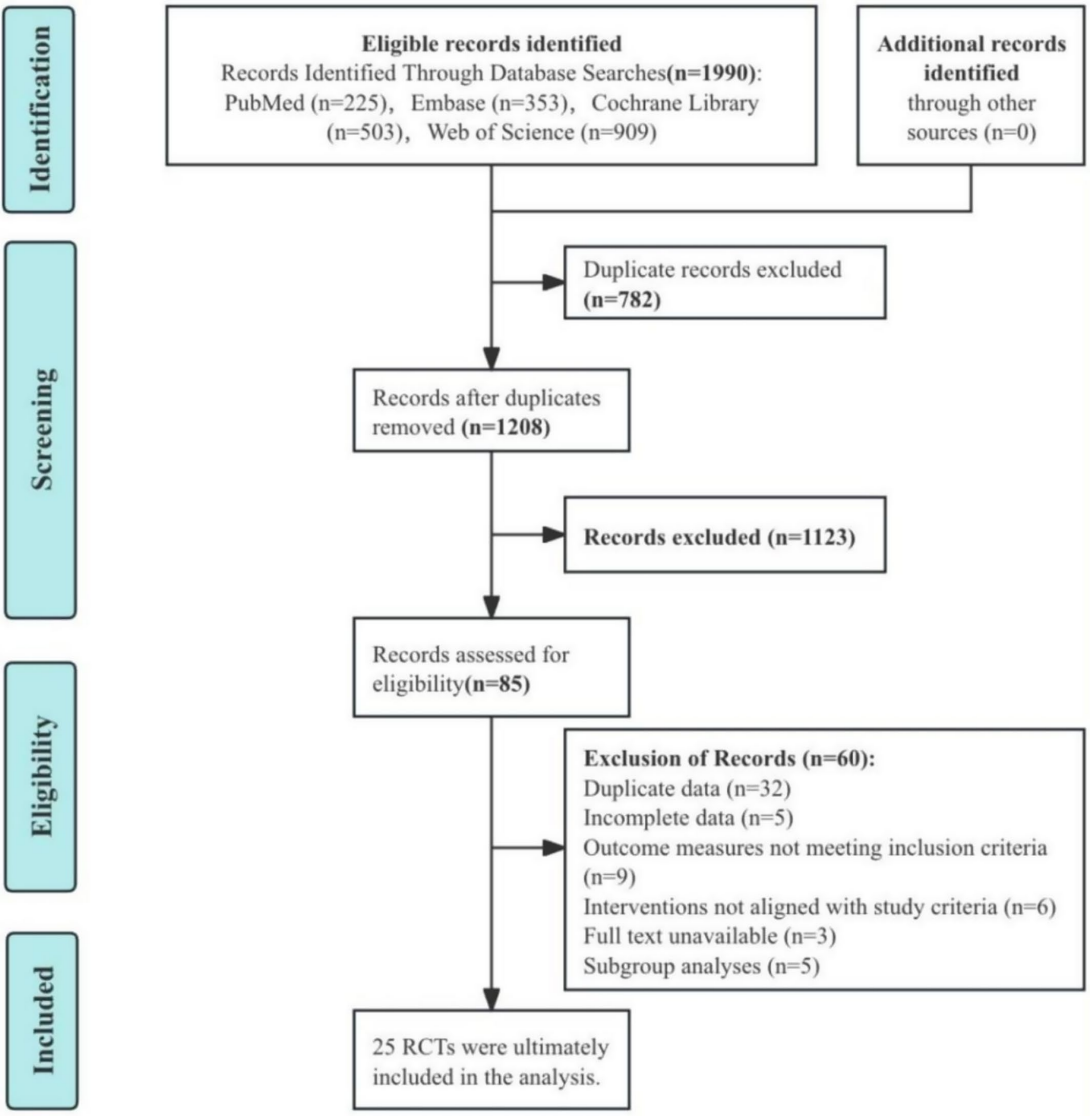
Flowchart of literature search and selection process.

A total of 1990 records were identified through database searches, including 225 from PubMed, 353 from Embase, 503 from the Cochrane Library, and 909 from the Web of Science. No additional records were identified through other sources. After removing 782 duplicates, 1,208 unique records were retained for screening. 1,123 records were excluded during the screening phase based on title and abstract screening. This left 85 records for full-text eligibility assessment. Of these, 60 records were excluded for the following reasons: 32 contained duplicate data, 5 had incomplete data, 9 lacked relevant outcome measures, 6 involved interventions that did not meet the study criteria, 3 lacked full-text availability, and 5 were subgroup analyses that were not eligible. Ultimately, 25 RCTs (22–46) met the inclusion criteria and were included in the final analysis. This process is summarized in Figure 1.

### Included study characteristics

The included trials involved 4,439 participants. The interventions included BIM, TRA and TAF for the treatment group and LAT for the control group. The mean age of patients varied from 22 to 94 years, except in 2 RCTs where the age was not specified. Sample sizes ranged from 60 to 661 participants. The treatment duration spanned from 3 to 12 months. Detailed information about the included studies is provided in Appendix 3 and Table S3.1. Further details on the four prostaglandin analogues evaluated in these trials are provided in Appendix 3 and Table S3.2.

### Risk of bias, certainty of evidence, and consistency

The risk of bias for each trial is outlined in Appendix 4. A major drawback was the insufficient details on blinding methods for participants and researchers. Among the 25 trials reviewed, 25 studies had a low risk of bias in random sequence generation, measurement of the outcome, and selection reporting. Additionally, 14 studies had a low risk of bias in deviations from intended interventions, and 22 had a low risk of missing outcome data. Overall, two studies had a high risk of bias, 6 raised concerns about potential bias, and 15 had a low risk. Our evaluation of the alignment between direct and indirect evidence showed strong consistency across all comparisons.

Furthermore, two outcomes did not reveal significant statistical evidence of global inconsistency. The τ^2^ results showed no significant heterogeneity within the network, with most comparisons displaying low to moderate heterogeneity levels (Appendix 5). The density, trace, and convergence diagnostic plots all showed strong convergence, confirming the robustness of the results (Appendices 6, 7). We evaluated the evidence quality with CINeMA and found that most pairwise comparisons had low to moderate confidence (Appendix 8). All networks adhered to the transitivity principle, ensuring the validity of indirect comparisons (Appendix 8; Table S8.1). Furthermore, we found no evidence of asymmetry in the funnel plots (Appendix 11).

### Intraocular pressure reduction

**Figure 2:**
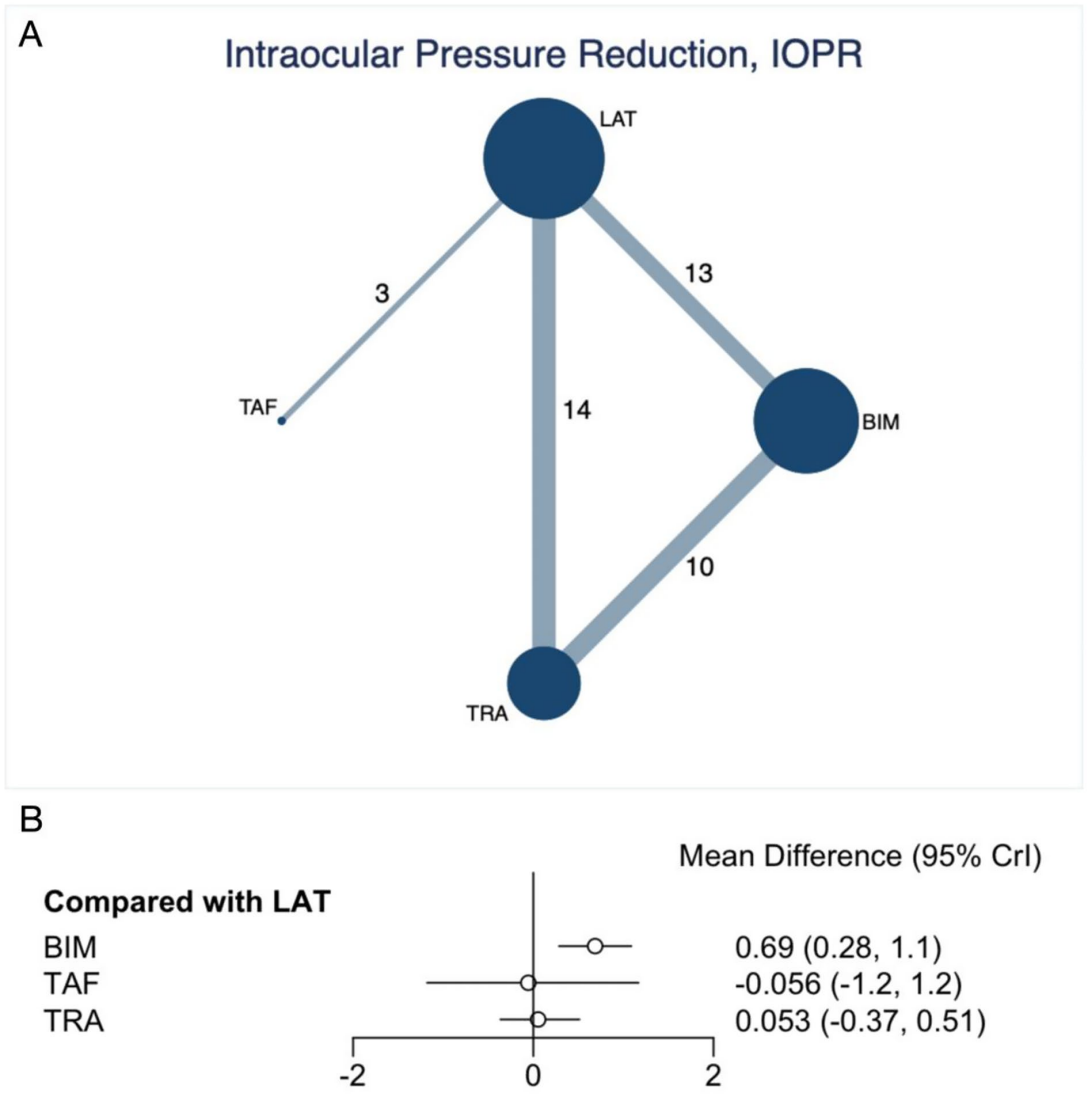
Network plot and forest plot of direct comparisons for IOPR. **(A)** Network of available comparisons of prostaglandins. The size of the nodes is proportional to the number of trial participants, and the thickness of the line connecting the nodes is proportional to the randomised number of trial participants directly comparing the two treatments. **(B)** Forest plot of network effect sizes between prostaglandins for IOPR.

Our NMA assessed the improvement in IOPR, encompassing 24 trials with 4,045 participants. As depicted in Figure 2A, the analysis included direct comparisons between prostaglandins. The thickness of the connecting lines indicates that prostaglandins were compared with each other. The forest plot displayed the direct comparison results among BIM, TAF, and TRA (Figure 2A). Specifically, BIM (MD 0.69, 95% CI 0.28 to 1.1, SUCRA 95.6%, moderate confidence of evidence) was associated with a significantly greater reduction in IOPR compared to LAT (Figure 2B; Appendix 9). Further comparisons of various prostaglandins revealed that BIM significantly outperformed TRA in improving the IOPR (MD 0.64, 95% CI 0.14 to 1.09, low confidence of evidence) (Appendix 10; Table S10.1). No statistically significant differences were observed in the comparisons among the other prostaglandins. According to CINeMA, the overall quality of evidence for IOPR was mainly rated as low to moderate (Appendix 8; Table S8.2).

### Incidence of conjunctival hyperemia

**Figure 3:**
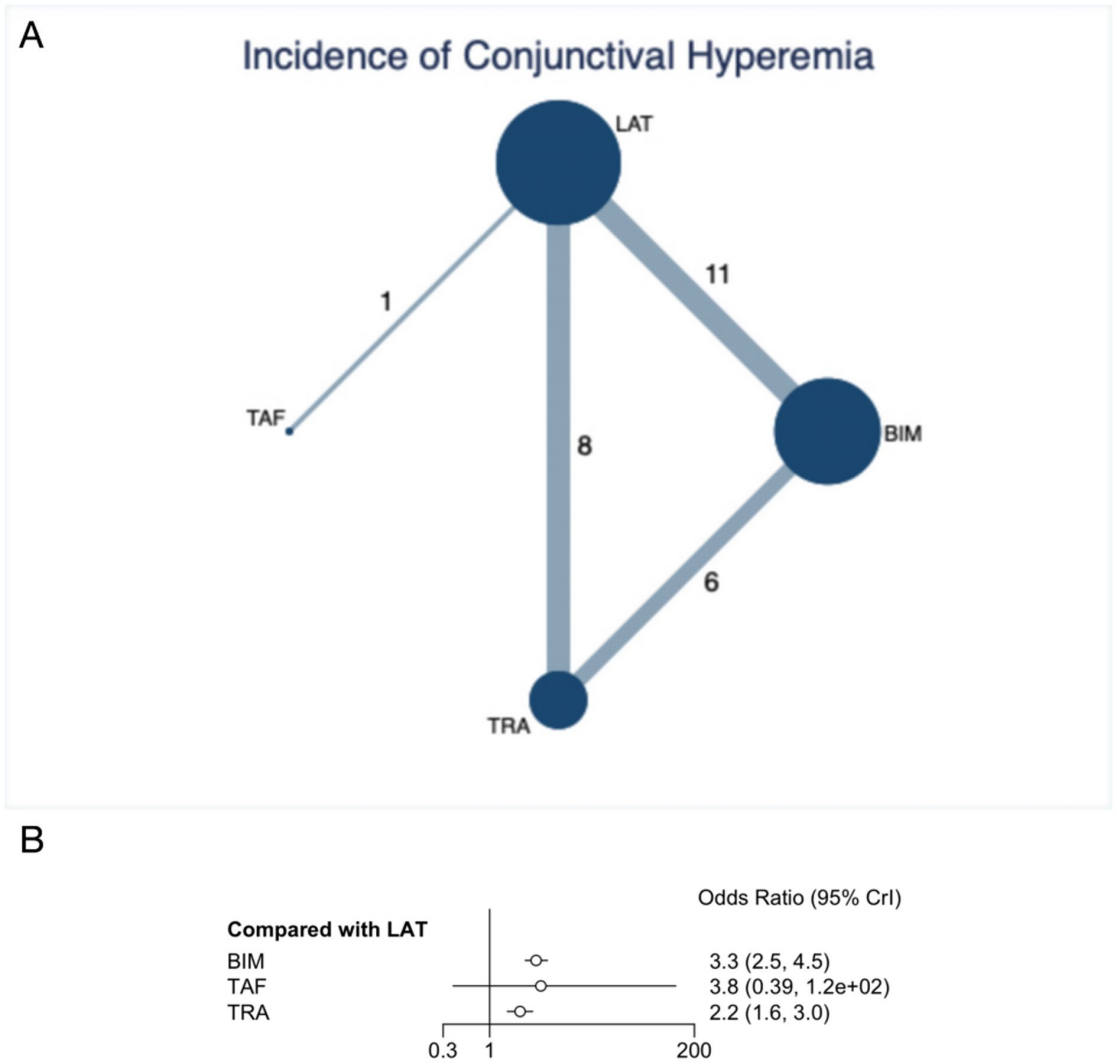
Network plot and forest plot of direct comparisons for incidence of conjunctival hyperemia. **(A)** Network of available comparisons of prostaglandins. The size of the nodes is proportional to the number of trial participants, and the thickness of the line connecting the nodes is proportional to the randomised number of trial participants directly comparing the two treatments. **(B)** Forest plot of network effect sizes between prostaglandins for incidence of conjunctival hyperemia.

The NMA on the incidence of conjunctival hyperemia included 16 RCTs involving 3,119 patients. Figure 3A presents the network plot, which directly compares prostaglandins. The thickness of the connecting lines highlights the more frequent comparisons between BIM and LAT. Figure 3 forest plot shows that BIM and TRA significantly increased the risk of conjunctival hyperemia. BIM, compared to LAT (OR 3.3, 95% CI 2.5 to 4.5, SUCRA 18.4%, high confidence of evidence), resulted in a significantly higher risk of conjunctival hyperemia. Similarly, TRA also showed a significantly increased risk of conjunctival hyperemia (OR 0.46, 95% CI 0.33 to 0.63, SUCRA 55%, high confidence of evidence) (Figure 3B; Appendix 9). Indirect comparisons indicated that BIM had a more significant risk on the incidence of conjunctival hyperemia than TRA (OR 1.51, 95% CI 1.06 to 2.16, high confidence of evidence) (Appendix 10; Table S10.2). Further comparisons of the incidence of conjunctival hyperemia are detailed in the SUCRA data (Appendix 9; Figure S9.2) and a table (Appendix 10; Table S10.2).

### Sensitivity analysis

To further examine the robustness of our findings, we performed sensitivity analyses by excluding studies at high risk of bias. As shown in Appendix 12, the results remained consistent with the primary analyses, supporting the robustness of our conclusions.

## Discussion

In this Bayesian network meta-analysis of 25 randomised controlled trials (4,045 participants) we found that, among the four licensed prostaglandin analogues for glaucoma or ocular hypertension, BIM produced the greatest additional reduction in intraocular pressure relative to LAT and was superior to TRA, whereas no other efficacy differences reached statistical significance. However, BIM and TRA were both associated with considerably higher odds of conjunctival hyperaemia than LAT, and indirect comparison showed BIM carried a higher risk than TRA. Overall, the certainty of evidence ranged from low to moderate for efficacy outcomes and was predominantly high for safety outcomes. These findings underscore the clinical trade-off between maximising pressure lowering and minimising ocular side effects when selecting a first-line prostaglandin analogue.

LAT, TRA, and TRA are PGF2α analogs that lower IOP by increasing aqueous humor outflow (47). In contrast, BIM has unique pharmacological properties. It is a synthetic prostamide that reduces IOP by stimulating FP prostaglandin receptors and is synthesized through cyclooxygenase-2 activity (48). This distinct mechanism may account for BIM’s superior IOP-lowering efficacy compared to other prostaglandin analogs.

Conjunctival hyperemia is the most common adverse effect associated with prostaglandin analogs (49). In this study, the incidence of conjunctival hyperemia was highest in the BIM group; however, no evidence from the included studies indicated an association with ocular surface inflammation. Preclinical data suggest that BIM, like other prostaglandin analogs, activates nitric oxide synthase, releasing nitric oxide and causing vasodilation (50). Moreover, multi-dose safety evaluations in rabbits, dogs, and non-human primates have shown that BIM does not induce or exacerbate conjunctival inflammation, suggesting that the hyperemia it causes is a non-inflammatory vasodilatory response (50).

Although local adverse reactions such as conjunctival hyperemia may negatively affect treatment adherence, evidence from two long-term open-label clinical studies indicates that hyperemia symptoms diminish significantly over time (51, 52). Therefore, discontinuing prostaglandin analogs solely due to conjunctival hyperemia is not recommended once IOP is effectively controlled.

In summary, effective clinical decision-making for patients with glaucoma and ocular hypertension requires a careful balance between therapeutic efficacy and tolerability. Based on our findings, BIM may be preferred in patients with advanced disease who require greater IOP reduction, while LAT may be more suitable for patients prioritizing tolerability. Treatment strategies should be individualized, taking into account the patient’s disease severity, risk tolerance, and preferences. Although prostaglandin analogues remain the first-line therapy, our analysis highlights the need for clearer guidance in choosing among them to optimize both efficacy and safety.

### Study limitations

This study has several limitations. First, although we conducted a comprehensive literature search, the possibility of publication bias cannot be fully excluded. Second, more than two-thirds of the included RCTs were conducted in high-income countries, with limited representation from low- and middle-income settings. This geographic imbalance may limit the generalisability of our findings to healthcare systems with different clinical practices, drug availability, and infrastructure. Third, estimates of intraocular pressure reduction were based exclusively on outcomes at 3 months, leaving the long-term efficacy of treatments uncertain. Finally, while this network meta-analysis offers robust comparative evidence by integrating data from multiple RCTs, its real-world applicability remains unclear. Differences in patient adherence, comorbidities, and treatment contexts are often underrepresented in trial populations. Real-world studies are needed to assess long-term effectiveness and tolerability across diverse clinical settings.

## Conclusion

This network meta-analysis provides comparative evidence on four prostaglandin analogues for glaucoma and ocular hypertension. BIM was associated with the greatest reduction in intraocular pressure, but also with a significantly higher risk of conjunctival hyperaemia. TRA and LAT showed more favourable safety profiles, albeit with modest efficacy. These results suggest a trade-off between pressure-lowering potency and tolerability.

## Data Availability

The original contributions presented in the study are included in the article/Supplementary material, further inquiries can be directed to the corresponding author.
