# Supplementary material for: Efficacy and safety of prostaglandin drugs for elevated intraocular pressure: a Bayesian network meta-analysis
Source: Front Med (Lausanne). 2025 Aug 11;12:1642986. doi: 10.3389/fmed.2025.1642986 (PMC12375679; doi:10.3389/fmed.2025.1642986)
Supplement: Supplementary file 1 [file Data_Sheet_1.docx]

**Supplementary Appendix**

**Efficacy and Safety of Prostaglandin Drugs for Elevated Intraocular Pressure: A Bayesian Network Meta-Analysis**

**Table of contents**

目录

[*Appendix 1: PRISMA NMA Checklist 2*](#_Toc12043)

[*Appendix 2: Search strategy 5*](#_Toc4191)

[*Appendix 3: Characteristics of included studies 7*](#_Toc26792)

[*Appendix 4: Risk of bias of randomized clinical trials 9*](#_Toc2094)

[*Appendix 5: Evaluation of Inconsistency and Heterogeneity 11*](#_Toc6382)

[*Appendix 6: Density plots and Trajectory plots of comparisons of each outcome 13*](#_Toc8040)

[*Appendix 7: Convergence Diagnostic Plot of comparisons of each outcome 15*](#_Toc3515)

[*Appendix 8: CINeMA Assessment 17*](#_Toc31150)

[*Appendix 9: SUCRA and cumulative probability plots 22*](#_Toc7298)

[*Appendix 10: League table of Summary Estimates for Prostaglandins on EMs Derived from Network Meta-analysis of 23 Trials 24*](#_Toc7602)

[*Appendix 11: Funnel plots 25*](#_Toc17418)

[*Appendix 12: Sensitivity analysis 26*](#_Toc24184)

**Appendix 1: PRISMA NMA Checklist**

| **Section/Topic** | **Item #** | **Checklist Item** | **Reported on Page #** |
| --- | --- | --- | --- |
| **TITLE** |  |  |  |
| Title | 1 | Identify the report as a systematic review *incorporating a network meta-analysis (or related form of meta-analysis).* | ***1*** |
|  |  |  |  |
| **ABSTRACT** |  |  |  |
| Structured summary | 2 | Provide a structured summary including, as applicable:  **Background:** main objectives  **Methods:** data sources; study eligibility criteria, participants, and interventions; study appraisal; and *synthesis methods, such as network meta-analysis.*  **Results:** number of studies and participants identified; summary estimates with corresponding confidence/credible intervals; *treatment rankings may also be discussed. Authors may choose to summarize pairwise comparisons against a chosen treatment included in their analyses for brevity.*  **Discussion/Conclusions:** limitations; conclusions and implications of findings.  **Other:** primary source of funding; systematic review registration number with registry name. | 1 |
|  |  |  |  |
| **INTRODUCTION** |  |  |  |
| Rationale | 3 | Describe the rationale for the review in the context of what is already known*, including mention of why a network meta-analysis has been conducted.* | ***2*** |
| Objectives | 4 | Provide an explicit statement of questions being addressed, with reference to participants, interventions, comparisons, outcomes, and study design (PICOS). | 2 |
|  |  |  |  |
| **METHODS** |  |  |  |
| Protocol and registration | 5 | Indicate whether a review protocol exists and if and where it can be accessed (e.g., Web address); and, if available, provide registration information, including registration number. | 3 |
| Eligibility criteria | 6 | Specify study characteristics (e.g., PICOS, length of follow-up) and report characteristics (e.g., years considered, language, publication status) used as criteria for eligibility, giving rationale. *Clearly describe eligible treatments included in the treatment network, and note whether any have been clustered or merged into the same node (with justification).* | ***3*** |
| Information sources | 7 | Describe all information sources (e.g., databases with dates of coverage, contact with study authors to identify additional studies) in the search and date last searched. | 4 |
| Search | 8 | Present full electronic search strategy for at least one database, including any limits used, such that it could be repeated. | 4 |
| Study selection | 9 | State the process for selecting studies (i.e., screening, eligibility, included in systematic review, and, if applicable, included in the meta-analysis). | 4 |
| Data collection process | 10 | Describe method of data extraction from reports (e.g., piloted forms, independently, in duplicate) and any processes for obtaining and confirming data from investigators. | 4 |
| Data items | 11 | List and define all variables for which data were sought (e.g., PICOS, funding sources) and any assumptions and simplifications made. | 3 |
| **Geometry of the network** | **S1** | Describe methods used to explore the geometry of the treatment network under study and potential biases related to it. This should include how the evidence base has been graphically summarized for presentation, and what characteristics were compiled and used to describe the evidence base to readers. | ***5*** |
| Risk of bias within individual studies | 12 | Describe methods used for assessing risk of bias of individual studies (including specification of whether this was done at the study or outcome level), and how this information is to be used in any data synthesis. | 4 |
| Summary measures | 13 | State the principal summary measures (e.g., risk ratio, difference in means). *Also describe the use of additional summary measures assessed, such as treatment rankings and surface under the cumulative ranking curve (SUCRA) values, as well as modified approaches used to present summary findings from meta-analyses.* | 5 |
| Planned methods of analysis | 14 | Describe the methods of handling data and combining results of studies for each network meta-analysis. This should include, but not be limited to:   - *Handling of multi-arm trials;* - *Selection of variance structure;* - *Selection of prior distributions in Bayesian analyses; and* - *Assessment of model fit.* | 5 |
| **Assessment of Inconsistency** | **S2** | Describe the statistical methods used to evaluate the agreement of direct and indirect evidence in the treatment network(s) studied. Describe efforts taken to address its presence when found. | 5 |
| Risk of bias across studies | 15 | Specify any assessment of risk of bias that may affect the cumulative evidence (e.g., publication bias, selective reporting within studies). | **4** |
| Additional analyses | 16 | Describe methods of additional analyses if done, indicating which were pre-specified. This may include, but not be limited to, the following:   - Sensitivity or subgroup analyses; - Meta-regression analyses; - *Alternative formulations of the treatment network; and* - *Use of alternative prior distributions for Bayesian analyses (if applicable).* | ***5*** |
| **RESULTS†** |  |  |  |
| Study selection | 17 | Give numbers of studies screened, assessed for eligibility, and included in the review, with reasons for exclusions at each stage, ideally with a flow diagram. | 6 |
| **Presentation of network structure** | **S3** | Provide a network graph of the included studies to enable visualization of the geometry of the treatment network. | ***8*** |
| **Summary of network geometry** | **S4** | Provide a brief overview of characteristics of the treatment network. This may include commentary on the abundance of trials and randomized patients for the different interventions and pairwise comparisons in the network, gaps of evidence in the treatment network, and potential biases reflected by the network structure. | ***8*** |
| Study characteristics | 18 | For each study, present characteristics for which data were extracted (e.g., study size, PICOS, follow-up period) and provide the citations. | 7 |
| Risk of bias within studies | 19 | Present data on risk of bias of each study and, if available, any outcome level assessment. | 7 |
| Results of individual studies | 20 | For all outcomes considered (benefits or harms), present, for each study: 1) simple summary data for each intervention group, and 2) effect estimates and confidence intervals. *Modified approaches may be needed to deal with information from larger networks.* | 7 |
| Synthesis of results | 21 | Present results of each meta-analysis done, including confidence/credible intervals. *In larger networks, authors may focus on comparisons versus a particular comparator (e.g. placebo or standard care), with full findings presented in an appendix. League tables and forest plots may be considered to summarize pairwise comparisons.* If additional summary measures were explored (such as treatment rankings), these should also be presented. | 7 |
| **Exploration for inconsistency** | **S5** | Describe results from investigations of inconsistency. This may include such information as measures of model fit to compare consistency and inconsistency models, *P* values from statistical tests, or summary of inconsistency estimates from different parts of the treatment network. | ***7*** |
| Risk of bias across studies | 22 | Present results of any assessment of risk of bias across studies for the evidence base being studied. | 7 |
| Results of additional analyses | 23 | Give results of additional analyses, if done (e.g., sensitivity or subgroup analyses, meta-regression analyses*, alternative network geometries studied, alternative choice of prior distributions for Bayesian analyses,* and so forth). | ***none*** |
| **DISCUSSION** |  |  |  |
| Summary of evidence | 24 | Summarize the main findings, including the strength of evidence for each main outcome; consider their relevance to key groups (e.g., healthcare providers, users, and policy-makers). | 10 |
| Limitations | 25 | Discuss limitations at study and outcome level (e.g., risk of bias), and at review level (e.g., incomplete retrieval of identified research, reporting bias). *Comment on the validity of the assumptions, such as transitivity and consistency. Comment on any concerns regarding network geometry (e.g., avoidance of certain comparisons).* | 11 |
| Conclusions | 26 | Provide a general interpretation of the results in the context of other evidence, and implications for future research. | 11 |
| **FUNDING** |  |  |  |
| Funding | 27 | Describe sources of funding for the systematic review and other support (e.g., supply of data); role of funders for the systematic review. This should also include information regarding whether funding has been received from manufacturers of treatments in the network and/or whether some of the authors are content experts with professional conflicts of interest that could affect use of treatments in the network. | ***none*** |

**Appendix 2: Search strategy**

**Table S1.** Search strategy of PubMed

| **#** | **Searches** |
| --- | --- |
| 1 | (((((((Ocular Hypertension[MeSH Terms]) OR (Hypertension, Ocular[Title/Abstract])) OR (Hypertensions, Ocular[Title/Abstract])) OR (Ocular Hypertensions[Title/Abstract])) OR (Glaucoma, Suspect[Title/Abstract])) OR (Glaucomas, Suspect[Title/Abstract])) OR (Suspect Glaucoma[Title/Abstract])) OR (Suspect Glaucomas[Title/Abstract]) OR Glaucoma[MeSH Terms]) |
| 2 | (((Prostaglandins[MeSH Terms]) OR (Prostanoids[Title/Abstract])) OR (Prostanoid[Title/Abstract])) OR (Prostaglandin[Title/Abstract]) |
| 3 | (((Latanoprost[MeSH Terms]) OR (PHXA41[Title/Abstract])) OR (PhXA34[Title/Abstract])) OR (Xalatan[Title/Abstract]) |
| 4 | ((((Bimatoprost[MeSH Terms]) OR (AGN 192024[Title/Abstract])) OR (192024, AGN[Title/Abstract])) OR (Lumigan[Title/Abstract])) OR (Latisse[Title/Abstract]) |
| 5 | ((((Travoprost[MeSH Terms]) OR ((((1R)-(1alpha(Z),2beta(1E,3R*),3alpha,5alpha))-7-(3,5-dihydroxy-2-(3-hydroxy-4-(3-trifluoromethyl)phenoxy)-1-butenyl)cyclopentyl)-5-heptenoic acid, 1-methylethyl ester[Title/Abstract])) OR (AL-6221[Title/Abstract])) OR (Travatan[Title/Abstract])) OR (Travatan Z[Title/Abstract]) |
| 6 | (tafluprost [Supplementary Concept]) OR (AFP-168[Title/Abstract]) |
| 7 | #2 OR #3 OR #4 OR #5 OR #6 |
| 8 | ("Randomized Controlled Trials as Topic"[MeSH] OR "Randomized Controlled Trial"[Title/Abstract] OR "Randomized Clinical Trial"[Title/Abstract] OR "RCT"[Title/Abstract] OR "Randomized Trial"[Title/Abstract] OR "Randomised Controlled Trial"[Title/Abstract] OR "Randomised Clinical Trial"[Title/Abstract]) |
| 9 | #1 AND #7 AND #8 |

**Table S2.** Search strategy of Web of Science

| **#** | **Searches** |
| --- | --- |
| 1 | TS=("Ocular Hypertension") OR TS=("Hypertension, Ocular") OR TS=("Hypertensions, Ocular") OR TS=("Ocular Hypertensions") OR TS=("Glaucoma, Suspect") OR TS=("Glaucomas, Suspect") OR TS=("Suspect Glaucoma") OR TS=("Suspect Glaucomas") OR TS=("Glaucoma") |
| 2 | TS=("Prostaglandins") OR TS=("Prostanoids") OR TS=("Prostanoid") OR TS=("Prostaglandin") |
| 3 | TS=("Latanoprost") OR TS=("PHXA41") OR TS=("PhXA34") OR TS=("Xalatan") |
| 4 | TS=("Bimatoprost") OR TS=("AGN 192024") OR TS=("192024, AGN") OR TS=("Lumigan") OR TS=("Latisse") |
| 5 | TS=("Travoprost") OR TS=("1R-(1alpha(Z),2beta(1E,3R*),3alpha,5alpha)-7-(3,5-dihydroxy-2-(3-hydroxy-4-(3-trifluoromethyl)phenoxy)-1-butenyl)cyclopentyl-5-heptenoic acid, 1-methylethyl ester") OR TS=("AL-6221") OR TS=("Travatan") OR TS=("Travatan Z") |
| 6 | TS=("Tafluprost") OR TS=("AFP-168") |
| 7 | #2 OR #3 OR #4 OR #5 OR #6 |
| 8 | TS=("Randomized Controlled Trials as Topic") OR TS=("Randomized Controlled Trial") OR TS=("Randomized Clinical Trial") OR TS=("RCT") OR TS=("Randomized Trial") OR TS=("Randomised Controlled Trial") OR TS=("Randomised Clinical Trial") |
| 9 | #1 AND #7 AND #8 |

**Table S3.** Search strategy of Cochrane Central Register of Controlled Trials

| **#** | **Searches** |
| --- | --- |
| 1 | "Ocular Hypertension" OR "Hypertension, Ocular" OR "Hypertensions, Ocular" OR "Ocular Hypertensions" OR "Glaucoma, Suspect" OR "Glaucomas, Suspect" OR "Suspect Glaucoma" OR "Suspect Glaucomas" OR “Glaucoma” |
| 2 | "Prostaglandins" OR "Prostanoids" OR "Prostanoid" OR "Prostaglandin" |
| 3 | "Latanoprost" OR "PHXA41" OR "PhXA34" OR "Xalatan" |
| 4 | "Bimatoprost" OR "AGN 192024" OR "192024, AGN" OR "Lumigan" OR "Latisse" |
| 5 | "Travoprost" OR "1R-(1alpha(Z),2beta(1E,3R*),3alpha,5alpha)-7-(3,5-dihydroxy-2-(3-hydroxy-4-(3-trifluoromethyl)phenoxy)-1-butenyl)cyclopentyl-5-heptenoic acid, 1-methylethyl ester" OR "AL-6221" OR "Travatan" OR "Travatan Z" |
| 6 | "Tafluprost" OR "AFP-168" |
| 7 | #2 OR #3 OR #4 OR #5 OR #6 |
| 8 | "Randomized Controlled Trials as Topic" OR "Randomized Controlled Trial" OR "Randomized Clinical Trial" OR "RCT" OR "Randomized Trial" OR "Randomised Controlled Trial" OR "Randomised Clinical Trial" |
| 9 | #1 AND #7 AND #8 |

**Table S4.** Search strategy of Embase

| **#** | **Searches** |
| --- | --- |
| 1 | ('ocular hypertension'/exp OR 'ocular hypertension':ti,ab OR 'hypertension, ocular':ti,ab OR 'ocular hypertensions':ti,ab OR 'hypertensions, ocular':ti,ab OR 'glaucoma, suspect':ti,ab OR 'suspect glaucoma':ti,ab OR 'suspect glaucomas':ti,ab OR 'glaucomas, suspect':ti,ab) OR 'Glaucoma'/exp |
| 2 | ('prostaglandin'/exp OR prostaglandins:ti,ab OR prostanoid:ti,ab OR prostanoids:ti,ab OR prostaglandin:ti,ab) |
| 3 | ('latanoprost'/exp OR latanoprost:ti,ab OR phxa41:ti,ab OR phxa34:ti,ab OR xalatan:ti,ab) |
| 4 | ('bimatoprost'/exp OR bimatoprost:ti,ab OR 'agn 192024':ti,ab OR '192024, agn':ti,ab OR lumigan:ti,ab OR latisse:ti,ab) |
| 5 | ('travoprost'/exp OR travoprost:ti,ab OR '1r-(1alpha(z),2beta(1e,3r*),3alpha,5alpha)-7-(3,5-dihydroxy-2-(3-hydroxy-4-(3-trifluoromethyl)phenoxy)-1-butenyl)cyclopentyl-5-heptenoic acid, 1-methylethyl ester':ti,ab OR al-6221:ti,ab OR travatan:ti,ab OR 'travatan z':ti,ab) |
| 6 | ('tafluprost'/exp OR tafluprost:ti,ab OR afp-168:ti,ab) |
| 7 | #2 OR #3 OR #4 OR #5 OR #6 |
| 8 | ('randomized controlled trial'/exp OR 'randomized controlled trial':ti,ab OR 'randomized clinical trial':ti,ab OR rct:ti,ab OR 'randomized trial':ti,ab OR 'randomised controlled trial':ti,ab OR 'randomised clinical trial':ti,ab) |
| 9 | #1 AND #7 AND #8 |

**Appendix 3: Characteristics of included studies**

**Table S3.1:** Baseline of characteristics of included studies

| **Study** | **Country** | **Study Design** | **Gender** | **Mean Age** | **Study Duration** | **Sample Size** | **Interventions** |
| --- | --- | --- | --- | --- | --- | --- | --- |
| Arcieri 2005 | Brazil | Single-Blind | 24/24 | 68 | 6 months | 15/16/17 | LAT/BIM/TRA |
| Birt 2010 | Canada | Single-Blind | 45/38 | 61 | 24 weeks | 30/27/26 | LAT/BIM/TRA |
| Cantor 2006 | USA | Single-Blind | 81/76 | 65 | 6 months | 76/81 | BIM/TRA |
| Cardascia 2003 | Italy | Double-Blind | 9/9 | 52 | 6 months | 9/9 | LAT/TRA |
| Cellini 2004 | Italy | Double-Blind | 32/28 | 64 | 6 months | 20/20/20 | LAT/BIM/TRA |
| Chiselita 2005 | Romania | Crossover Single-Blind | NA | 65 | 3 months | 38/38 | LAT/TRA |
| Faridi 2010 | USA | Single-Blind | 65/57 | 68 | 6 months | 42/40/40 | LAT/BIM/TRA |
| Fogagnolo 2015 | Italy | Single-Blind | 20/15 | 66 | 12 months | 20/20 | LAT/TAF |
| Gandolfi 2001 | Italy + USA | Double-Blind | 87/145 | 62 | 3 months | 113/119 | LAT/BIM |
| Hepsen 2007 | Turkey | Single-Blind | 20/25 | 62 | 3 months | 15/15/15 | LAT/BIM/TRA |
| Kim 2021 | South Korea | Single-Blind | 53/38 | 56 | 12 weeks | 46/45 | LAT/TAF |
| Konstas 2007 | Greece | Crossover Single-Blind | NA | 67 | 3 months | 129/129 | LAT/BIM |
| Mishra 2014 | Italy | Single-Blind | 54/51 | 54 | 12 months | 35/35/35 | LAT/BIM/TRA |
| Muz 2021 | Turkey | Single-Blind | 17/27 | 62 | 12 months | 22/22 | LAT/TRA |
| Nct 2007 | USA | Double-Blind | 241/302 | NA | 18 weeks | 272/270 | LAT/BIM |
| Netland 2001 | USA | Single-Blind | 189/201 | 22~94 | 12 months | 193/197 | LAT/TRA |
| Noecker 2003 | USA | Single-Blind | 11/20 | 65 | 3 months | 16/15 | BIM/TRA |
| Park 2015 | South Korea | NA | 18/23 | 55 | 3 months | 21/20 | LAT/TAF |
| Parmaksiz 2006 | Turkey | Single-Blind | NA | NA | 9 months | 16/18 | LAT/TRA |
| Parrish 2003 | USA | Single-Blind | 172/238 | 65 | 12 months | 136/136/138 | LAT/BIM/TRA |
| Stalmans 2016 | Europe | Crossover Single-Blind | NA | 39~85 | 6 months | 28/28 | LAT/BIM |
| Varma 2008 | USA | Single-Blind | 172/238 | 65 | 12 months | 136/136/138 | LAT/BIM/TRA |
| Whitson 2010 | USA | Single-Blind | 45/61 | 68.3 | 3 months | 38/35/33 | LAT/BIM/TRA |
| Walters 2022 | USA | Double-Blind | 193/240 | 18~85 | 6 months | 107/326 | LAT/BIM |
| Fechtner2024 | USA+China | Double-Blind | 273/388 | 63 | 3 months | 333/328 | LAT/BIM |

Note: Latanoprost (LAT); Bimatoprost (BIM); Travoprost (TRA); TAF (TAF); Not Available (NA).

Table S3.2: Prostaglandins list

| **Prostaglandins** | **Synonyms** | **Drug Type** | **Company** | **Highest Phase** | **Drug-Approved Country/Region** | **Anatomical Therapeutic Chemical** |
| --- | --- | --- | --- | --- | --- | --- |
| Latanoprost | Xalatan | Prostaglandin Analog | Pfizer | Approved | Global (e.g., USA, EU, Asia) | S01EE01 |
| Bimatoprost | Lumigan | Prostaglandin Analog | Allergan (AbbVie) | Approved | Global (e.g., USA, EU, Asia) | S01EE03 |
| Travoprost | Travatan | Prostaglandin Analog | Alcon | Approved | Global (e.g., USA, EU, Asia) | S01EE04 |
| TAF | Zioptan | Prostaglandin Analog | Santen | Approved | Global (e.g., USA, EU, Asia) | S01EE05 |

**Appendix 4: Risk of bias of randomized clinical trials**

**Figure S4:** Overall risk of bias presented as percentage of each risk of bias item across all included studies.

Green = Low risk, Red = High risk, Yellow = Some concerns.


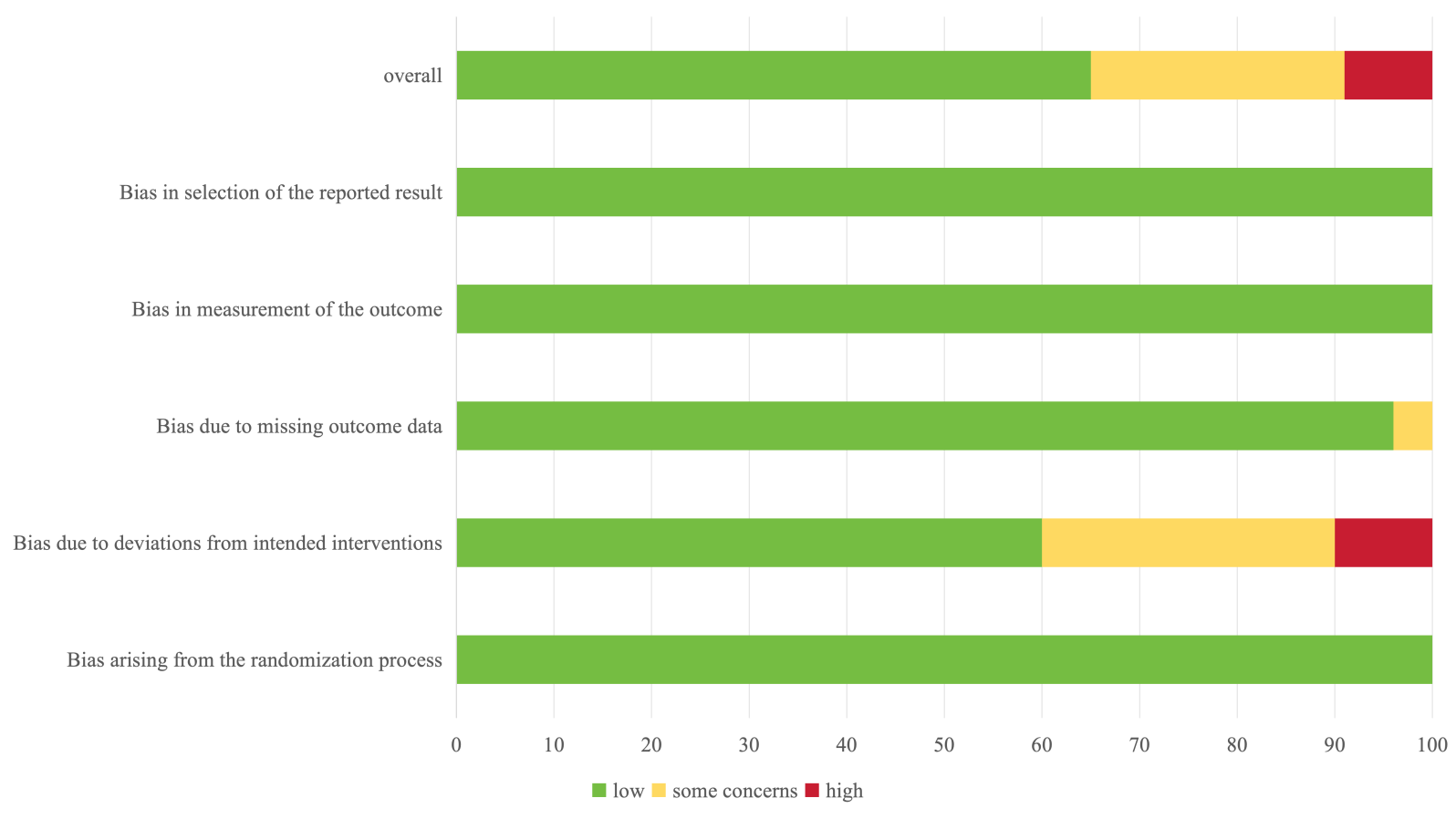


**Table S4:** Study level risk of bias assessment using Cochrane risk of bias tool 2.0 for assessing risk of bias of randomized clinical trials.

| Study | Randomization process | Deviations from intended interventions | Missing outcome data | Measurement of the outcome | Selection of the reported result | Overall |
| --- | --- | --- | --- | --- | --- | --- |
| Arcieri 2005 | low | low | low | low | low | low |
| Birt 2010 | low | some concerns | some concerns | low | low | high |
| Cantor 2006 | low | low | low | low | low | low |
| Cardascia 2003 | low | low | low | low | low | low |
| Cellini 2004 | low | some concerns | low | low | low | some concerns |
| Chiselita 2005 | low | some concerns | low | low | low | some concerns |
| Faridi 2010 | low | high | low | low | low | low |
| Fogagnolo 2015 | low | low | low | low | low | low |
| Gandolfi 2001 | low | low | low | low | low | low |
| Hepsen 2007 | low | some concerns | low | low | low | some concerns |
| Kim 2021 | low | some concerns | low | low | low | some concerns |
| Konstas 2007 | low | low | low | low | low | low |
| Mishra 2014 | low | low | low | low | low | low |
| Muz 2021 | low | some concerns | low | low | low | some concerns |
| Nct 2007 | low | some concerns | low | low | low | some concerns |
| Netland 2001 | low | low | low | low | low | low |
| Noecker 2003 | low | low | low | low | low | low |
| Park 2015 | low | low | low | low | low | low |
| Parmaksiz 2006 | low | low | low | low | low | low |
| Parrish 2003 | low | low | low | low | low | low |
| Stalmans 2016 | low | low | low | low | low | low |
| Varma 2008 | low | low | low | low | low | low |
| Whitson 2010 | low | high | low | low | low | high |
| Arcieri 2005 | low | low | low | low | low | low |

**Appendix 5: Evaluation of Inconsistency and Heterogeneity**

**Table S5.1: Global inconsistency and Heterogeneity**

| **Outcome** | **Consistency** | | | **Global inconsistency** | | | **Heterogeneity** |
| --- | --- | --- | --- | --- | --- | --- | --- |
|  | **Dbar** | **pD** | **DIC** | **Dbar** | **pD** | **DIC** | **τ²** |
| **Intraocular Pressure Reduction** | 58.28733 | 39.08119 | 97.36852 | 58.52833 | 39.79625 | 98.32458 | 0.2681686 |
| **Incidence of Conjunctival Hyperemia** | 32.4186 | 22.13391 | 54.5525 | 31.98315 | 22.85628 | 54.83943 | 0.03115225 |

**Table S5.2: Side-splitting of Intraocular Pressure Reduction. Inconsistency test between direct and indirect treatment comparisons in mixed treatment comparison.**


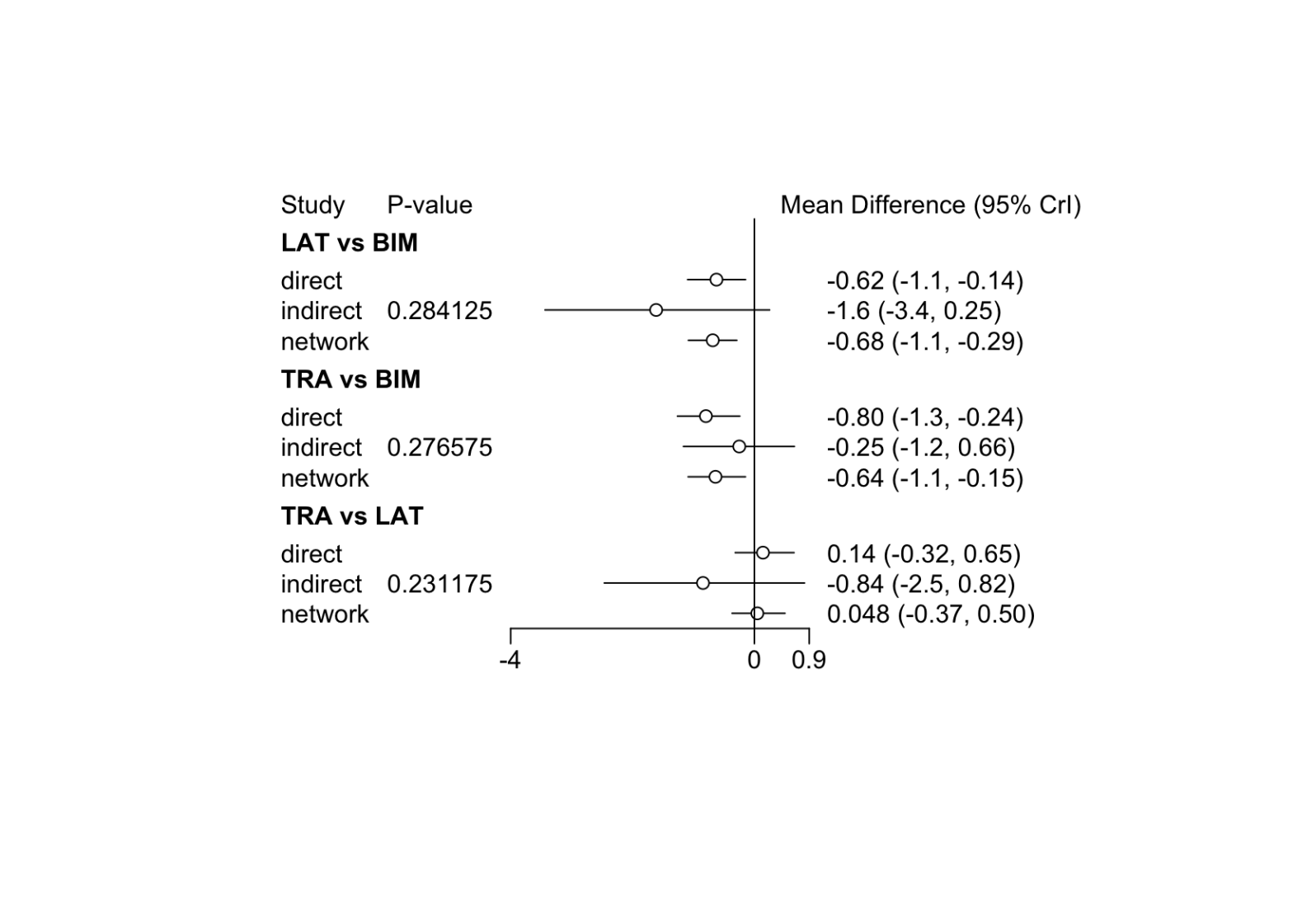


**Table S5.3: Side-splitting of Incidence of Conjunctival Hyperemia. Inconsistency test between direct and indirect treatment comparisons in mixed treatment comparison.**


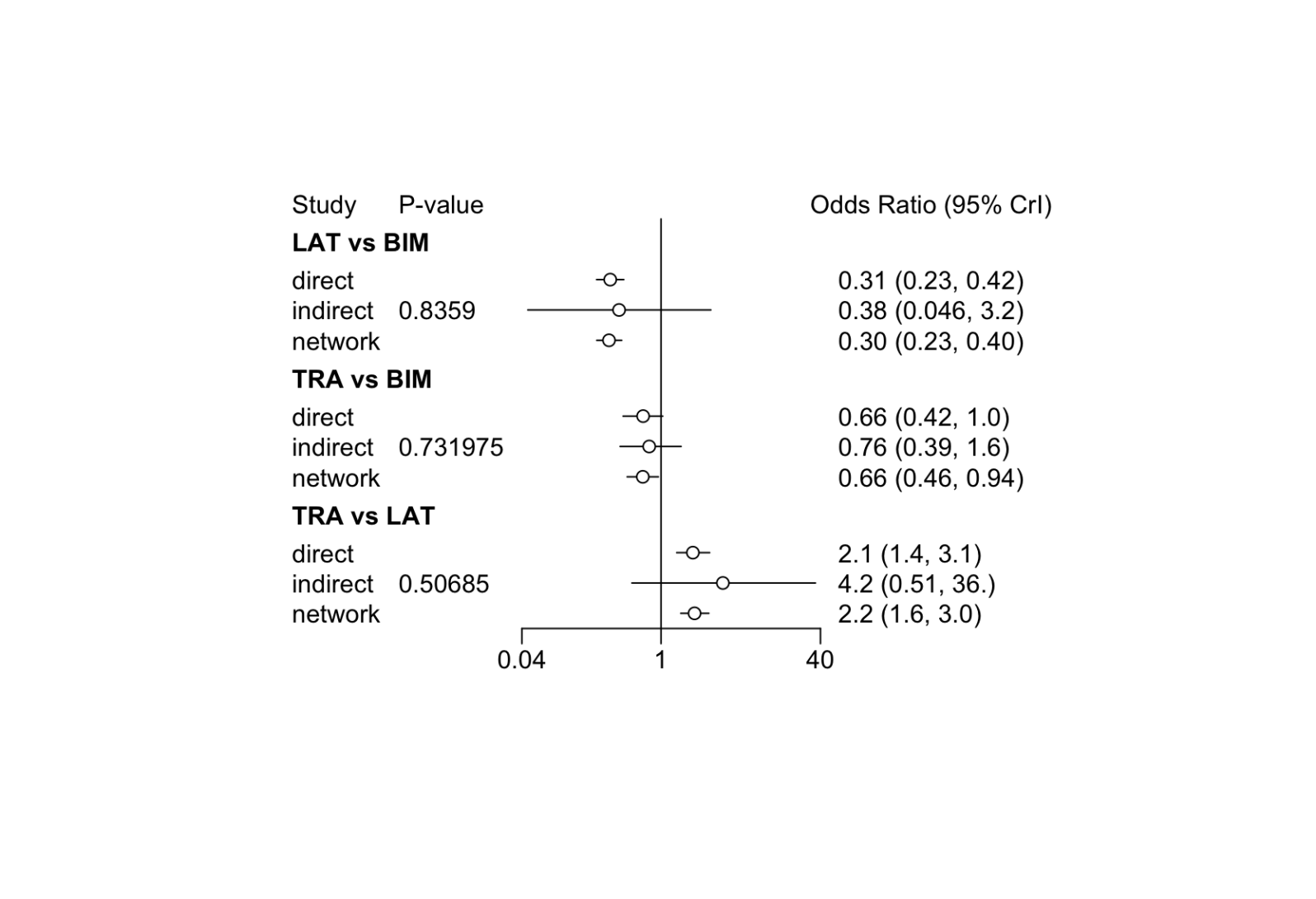


**Appendix 6: Density plots and Trajectory plots of comparisons of each outcome**

**Figure S6.1:** Density plots and Trajectory plots of **Intraocular Pressure Reduction**

**
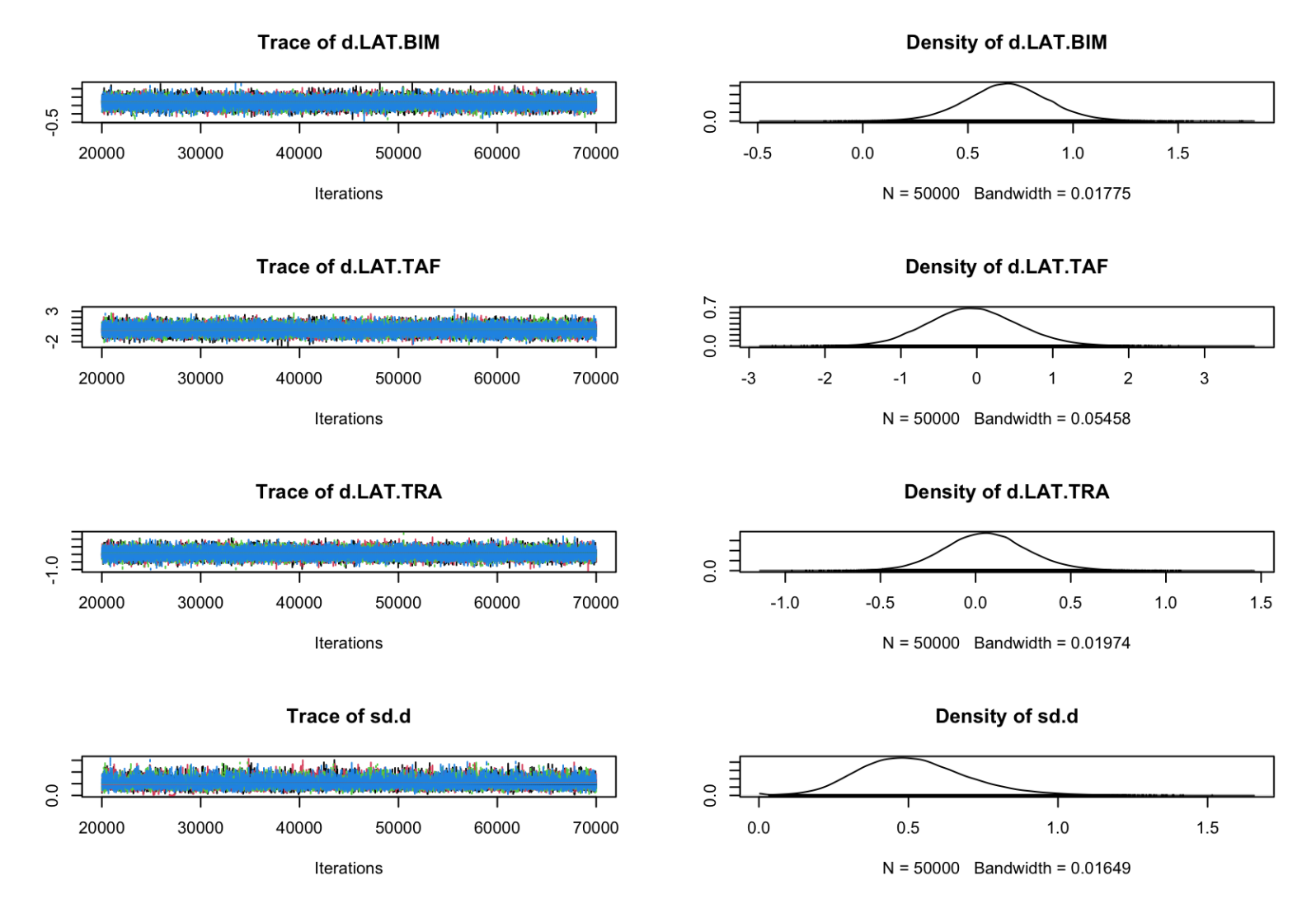
**

**Figure S6.2:** Density plots and Trajectory plots of **Incidence of Conjunctival Hyperemia**

**
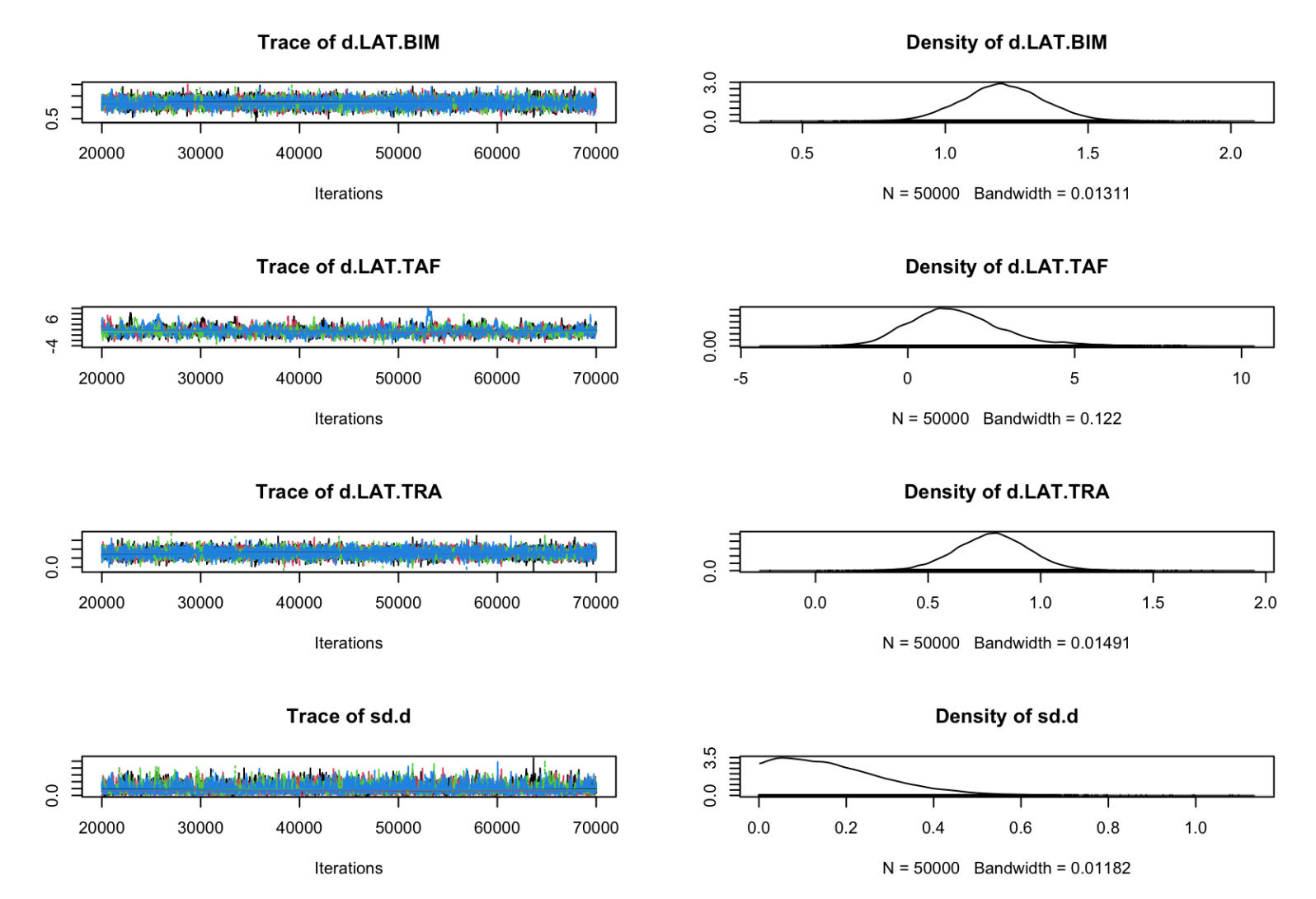
**

**Appendix 7: Convergence Diagnostic Plot of comparisons of each outcome**

**Figure S7.1:** Convergence Diagnostic Plot of **Intraocular Pressure Reduction**

**
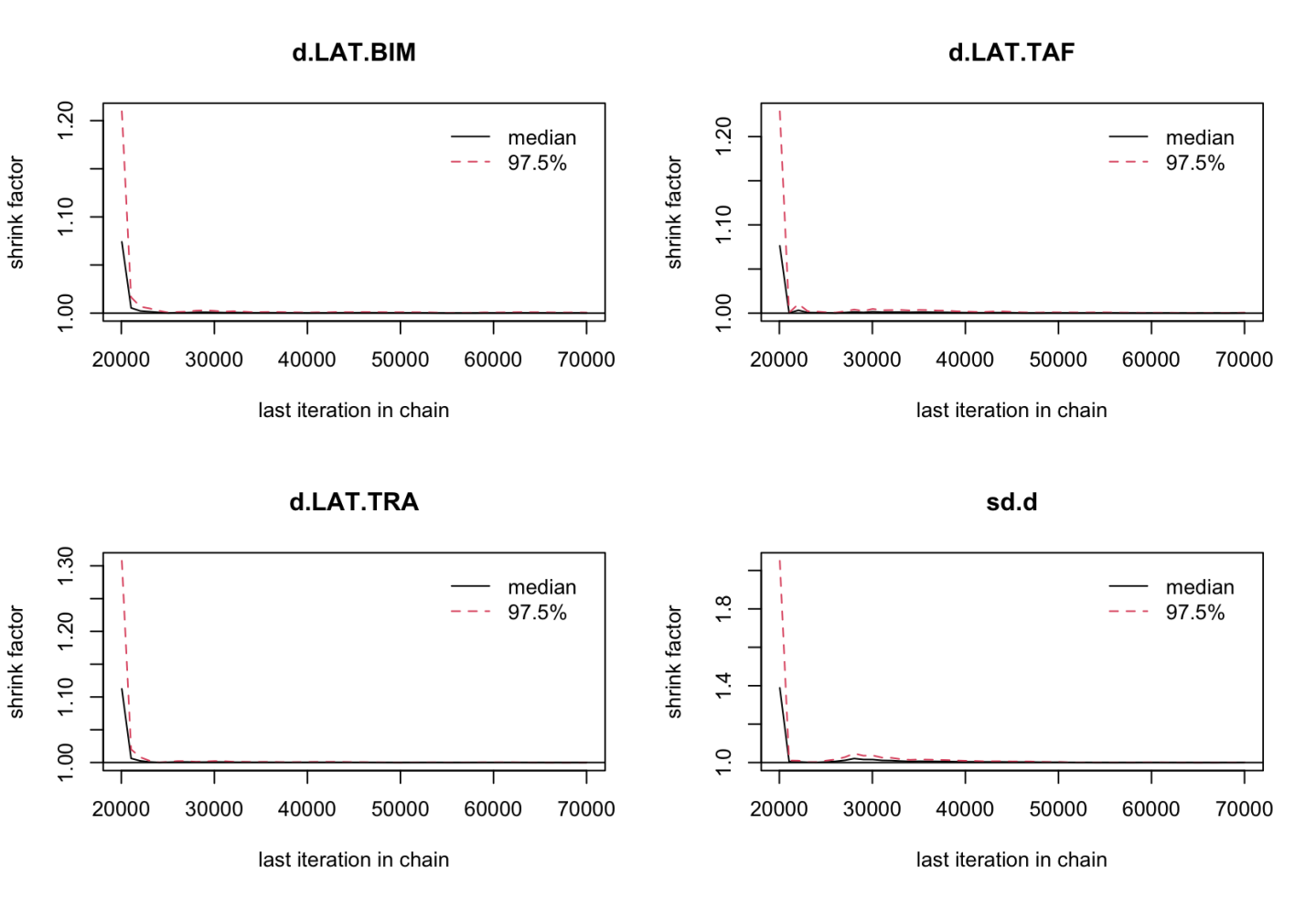
**

**Figure S7.2:** Convergence Diagnostic Plot of **Incidence of Conjunctival Hyperemia**

**
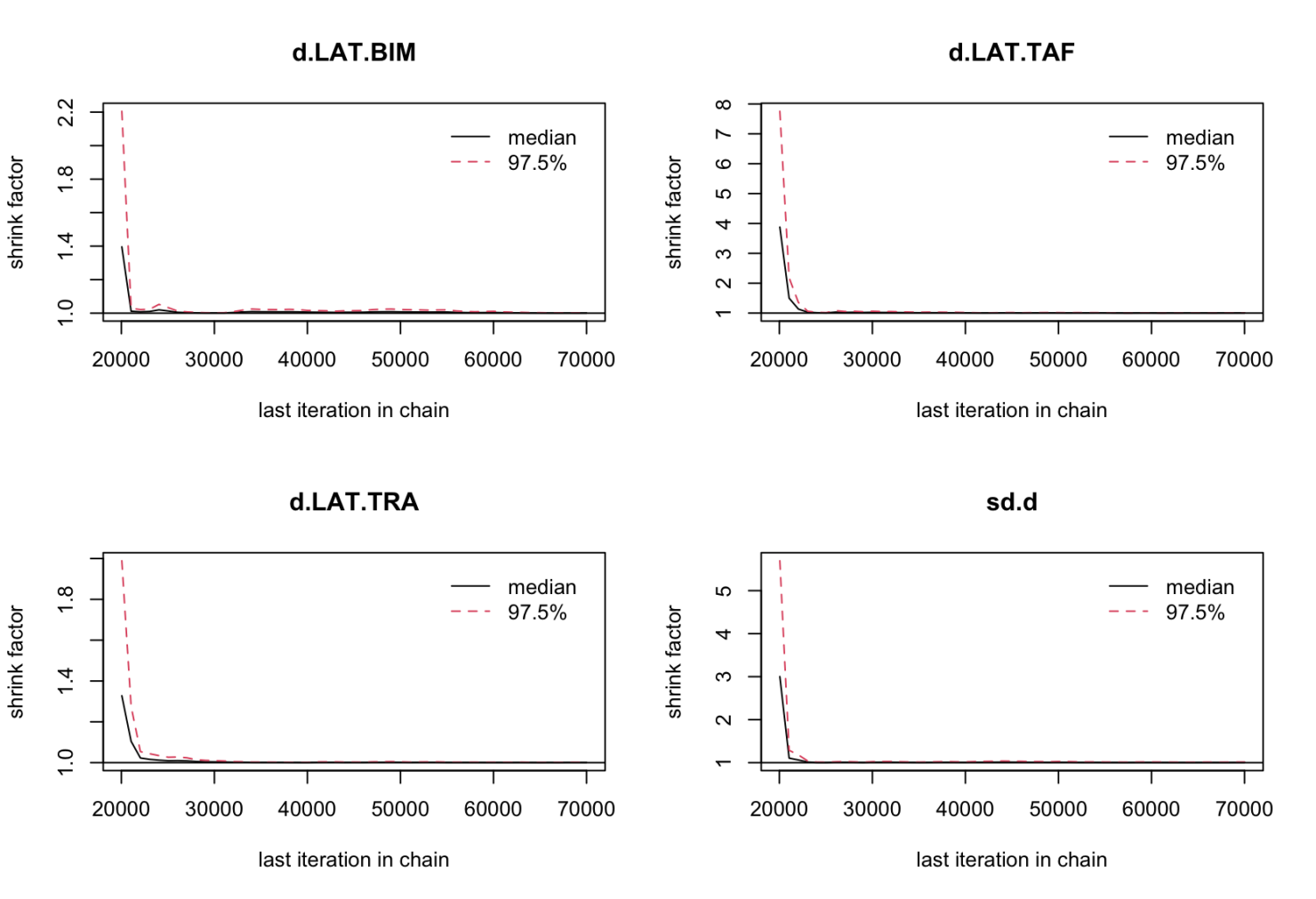
**

**Appendix 8: CINeMA Assessment**

We use the CINeMA framework to evidence certainty, assessing it for each network estimate based on the following criteria:

- **Within study bias:** We classified the overall risk of bias for each study as low, moderate, or high when none of the four assessed risk of bias items were rated as high risk. See Appendix 4 for the bias assessment. The risk of bias for pairwise comparison of each drug is shown in **Figure S8.1-8.2.**

**Figure S8.1:** Risk of bias contribution by intervention group in **Intraocular Pressure Reduction**

**
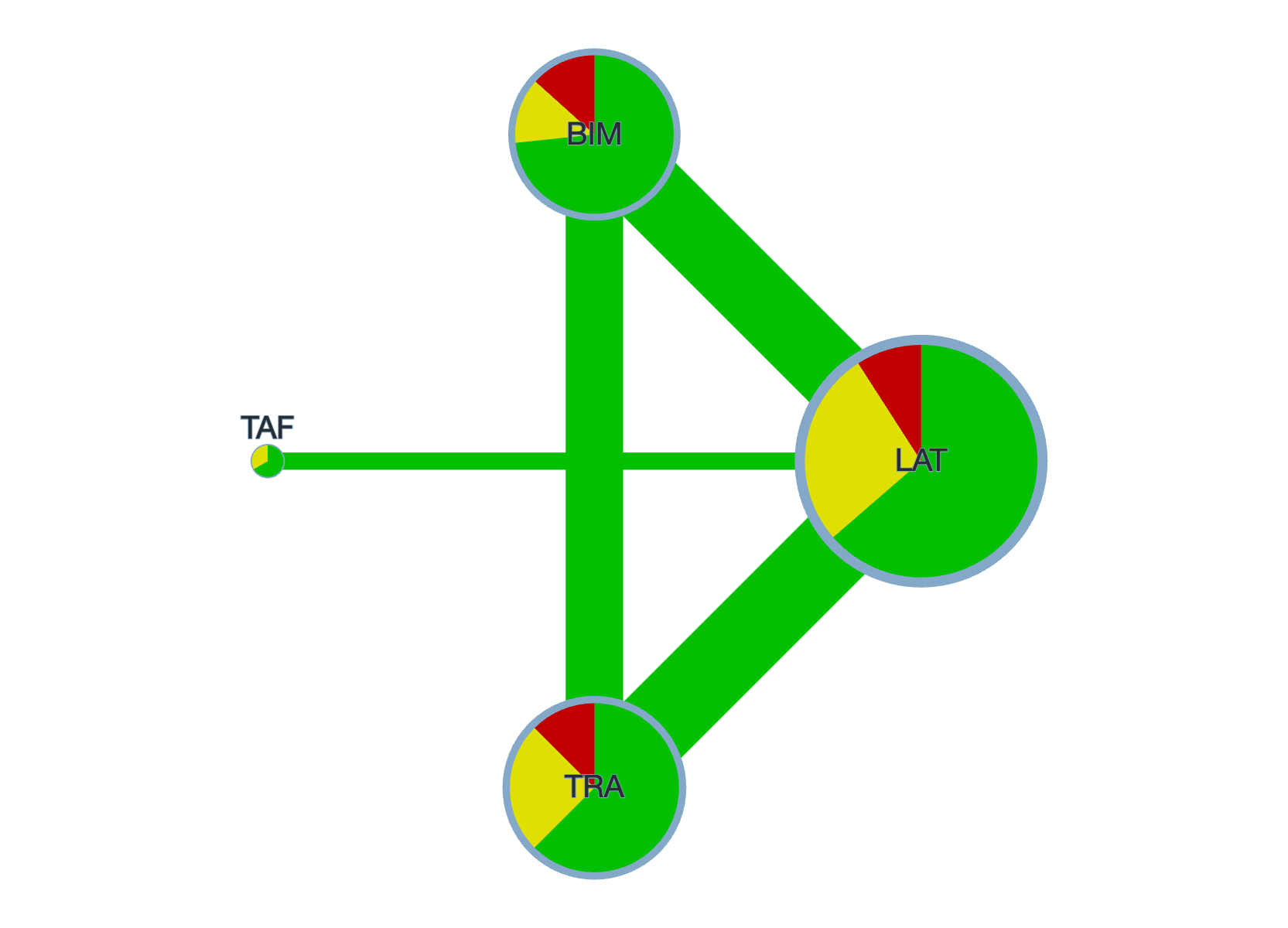
**

**Figure S8.2:** Overall risk of bias by treatment comparison in **Intraocular Pressure Reduction**

**
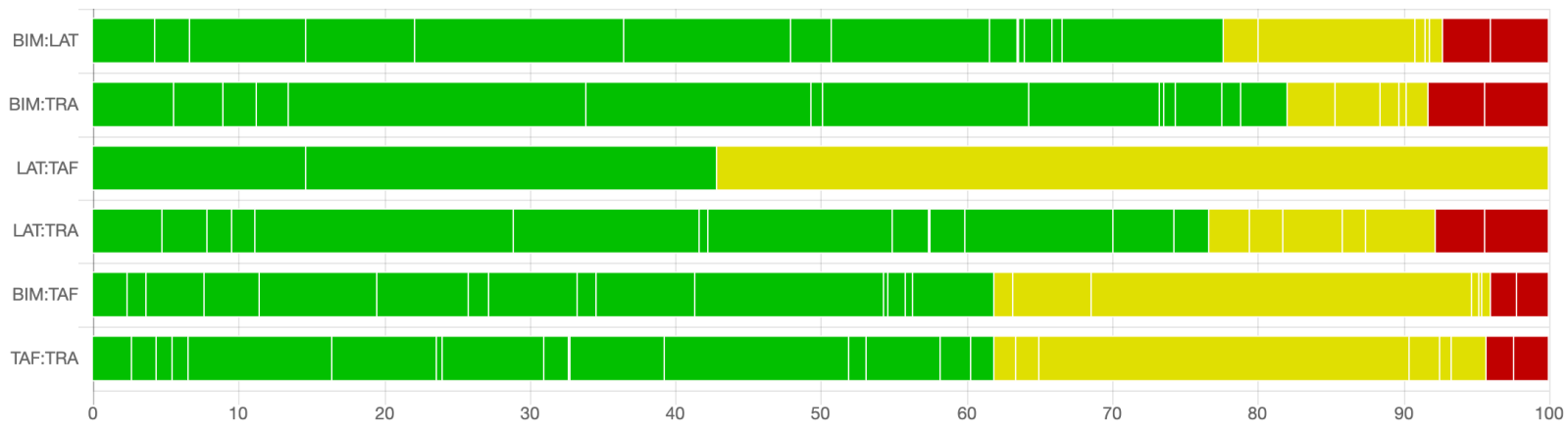
**

**Figure S8.3:** Risk of bias contribution by intervention group in **Incidence of Conjunctival Hyperemia**

**
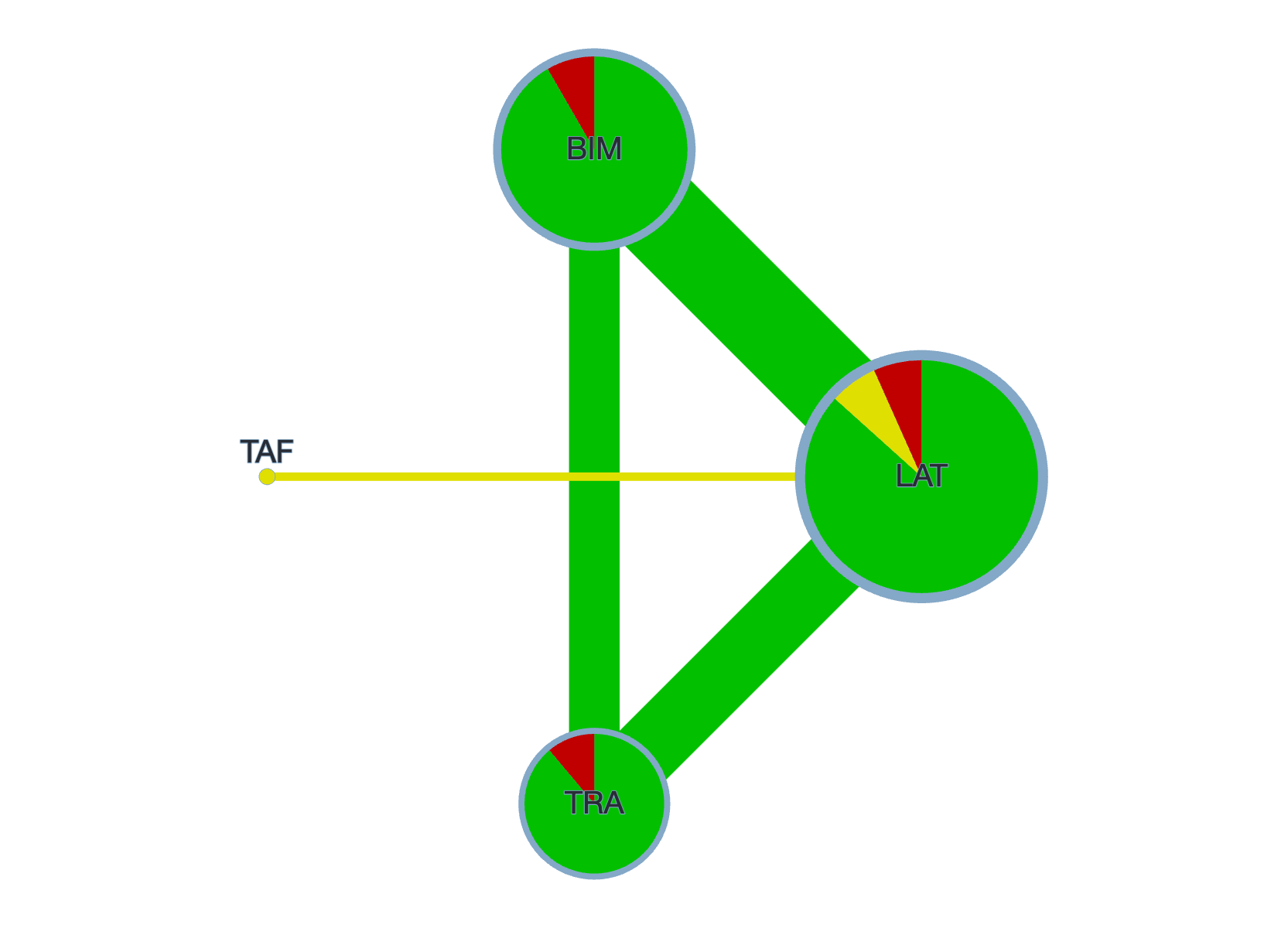
**

**Figure S8.4:** Overall risk of bias by treatment comparison in **Incidence of Conjunctival Hyperemia**

**
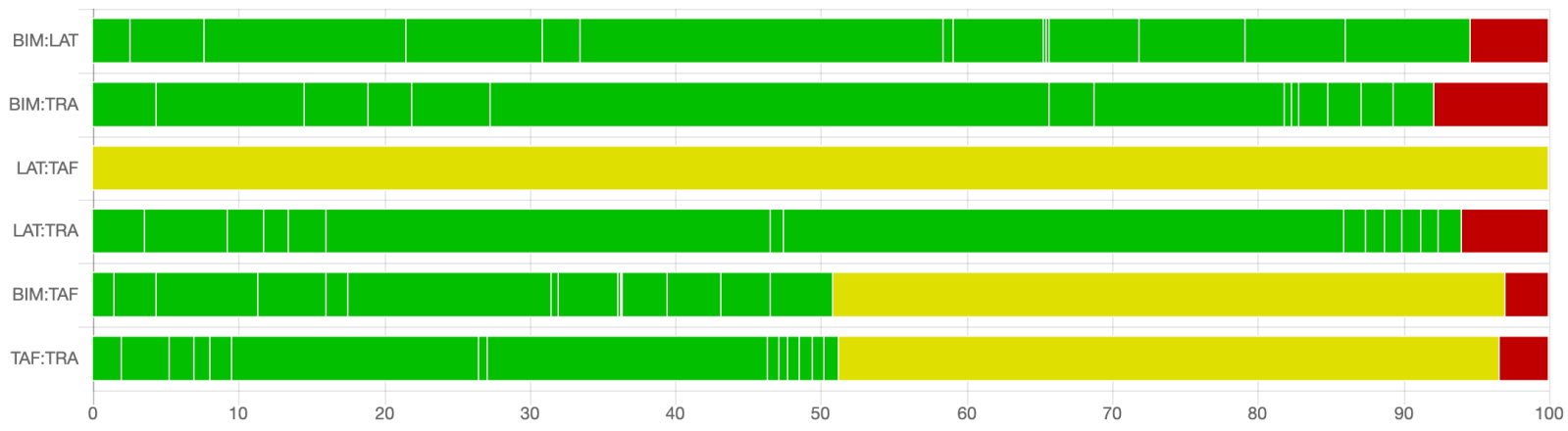
**

- **Reporting bias:** We judged it using Bayesian diagnostics, including MCMC convergence (trace plots, Gelman-Rubin statistic), posterior density plots, and sensitivity analyses **(Appendix 6-7)**.
- **Indirectness:** Transferability assumptions were assessed by reporting the mean total prior miscarriages in the included study population and by comparing age at baseline concordance between groups.

**Table S8.1:** Transitivity (Indirectness) Assessment

| **Study** | **Study Design** | **Mean Age** | **Study Duration** |
| --- | --- | --- | --- |
| Arcieri 2005 | Single-Blind | 68 | 6 months |
| Birt 2010 | Single-Blind | 61 | 24 weeks |
| Cantor 2006 | Single-Blind | 65 | 6 months |
| Cardascia 2003 | Double-Blind | 52 | 6 months |
| Cellini 2004 | Double-Blind | 64 | 6 months |
| Chiselita 2005 | Crossover Single-Blind | 65 | 3 months |
| Faridi 2010 | Single-Blind | 68 | 6 months |
| Fogagnolo 2015 | Single-Blind | 66 | 12 months |
| Gandolfi 2001 | Double-Blind | 62 | 3 months |
| Hepsen 2007 | Single-Blind | 62 | 3 months |
| Kim 2021 | Single-Blind | 56 | 12 weeks |
| Konstas 2007 | Crossover Single-Blind | 67 | 3 months |
| Mishra 2014 | Single-Blind | 54 | 12 months |
| Muz 2021 | Single-Blind | 62 | 12 months |
| Nct 2007 | Double-Blind | NA | 18 weeks |
| Netland 2001 | Single-Blind | 22~94 | 12 months |
| Noecker 2003 | Single-Blind | 65 | 3 months |
| Park 2015 | NA | 55 | 3 months |
| Parmaksiz 2006 | Single-Blind | NA | 9 months |
| Parrish 2003 | Single-Blind | 65 | 12 months |
| Stalmans 2016 | Crossover Single-Blind | 39~85 | 6 months |
| Varma 2008 | Single-Blind | 65 | 12 months |
| Whitson 2010 | Single-Blind | 68.3 | 3 months |
| Walters 2022 | Double-Blind | 18~85 | 6 months |
| Fechtner 2024 | Double-Blind | 63 | 3 months |

- **Imprecision:** We use the CINeMA website to grade the accuracy of each comparison.
- **Heterogeneity:** We assessed the degree of worry by comparing clinical reasoning based on 95% confidence intervals (CIs) while applying the same clinical reasoning framework as for inaccuracy. In particular, we judged the consistency of our findings based on the confidence and prediction intervals associated with clinically important effect sizes. And we used the same thresholds of clinical significance as described above and followed the recommendations automatically provided by CINeMA (https://cinema.ispm.unibe.ch/).
- **Inconsistency:** For inconsistency, we looked at the results for node splitting **(Appendix 5)** and we saw major problems when p<0.10, but otherwise no problems.

**Table S8.2:** CINeMA Results of **Intraocular Pressure Reduction**

| Comparison | Within-study bias | Reporting bias | Indirectness | Imprecision | Heterogeneity | Incoherence | Confidence rating |
| --- | --- | --- | --- | --- | --- | --- | --- |
| **BIM:LAT** | No concerns | Low risk | No concerns | No concerns | Some concerns | No concerns | Moderate |
| **BIM:TRA** | No concerns | Low risk | No concerns | No concerns | Major concerns | No concerns | Low |
| **LAT:TAF** | Some concerns | Low risk | Some concerns | Major concerns | No concerns | No concerns | Low |
| **LAT:TRA** | No concerns | Low risk | No concerns | Major concerns | No concerns | No concerns | Low |
| **BIM:TAF** | No concerns | Low risk | No concerns | Major concerns | No concerns | No concerns | Low |
| **TAF:TRA** | No concerns | Low risk | No concerns | Major concerns | No concerns | No concerns | Low |

**Table S8.2:** CINeMA Results of **Incidence of Conjunctival Hyperemia**

| Comparison | Within-study bias | Reporting bias | Indirectness | Imprecision | Heterogeneity | Incoherence | Confidence rating |
| --- | --- | --- | --- | --- | --- | --- | --- |
| **BIM:LAT** | No concerns | Low risk | No concerns | No concerns | No concerns | No concerns | High |
| **BIM:TRA** | No concerns | Low risk | No concerns | No concerns | No concerns | No concerns | High |
| **LAT:TAF** | Some concerns | Low risk | No concerns | Major concerns | No concerns | No concerns | Low |
| **LAT:TRA** | No concerns | Low risk | No concerns | No concerns | No concerns | No concerns | High |
| **BIM:TAF** | No concerns | Low risk | No concerns | Major concerns | No concerns | No concerns | Low |
| **TAF:TRA** | No concerns | Low risk | No concerns | Major concerns | No concerns | No concerns | Low |

**Appendix 9: SUCRA and cumulative probability plots**

**Figure S9.1**: Cumulative ranking curve plots of Prostaglandins for **Intraocular Pressure Reduction** in range network. Higher surface under the curve reflects higher probability of association with **Intraocular Pressure Reduction**

**
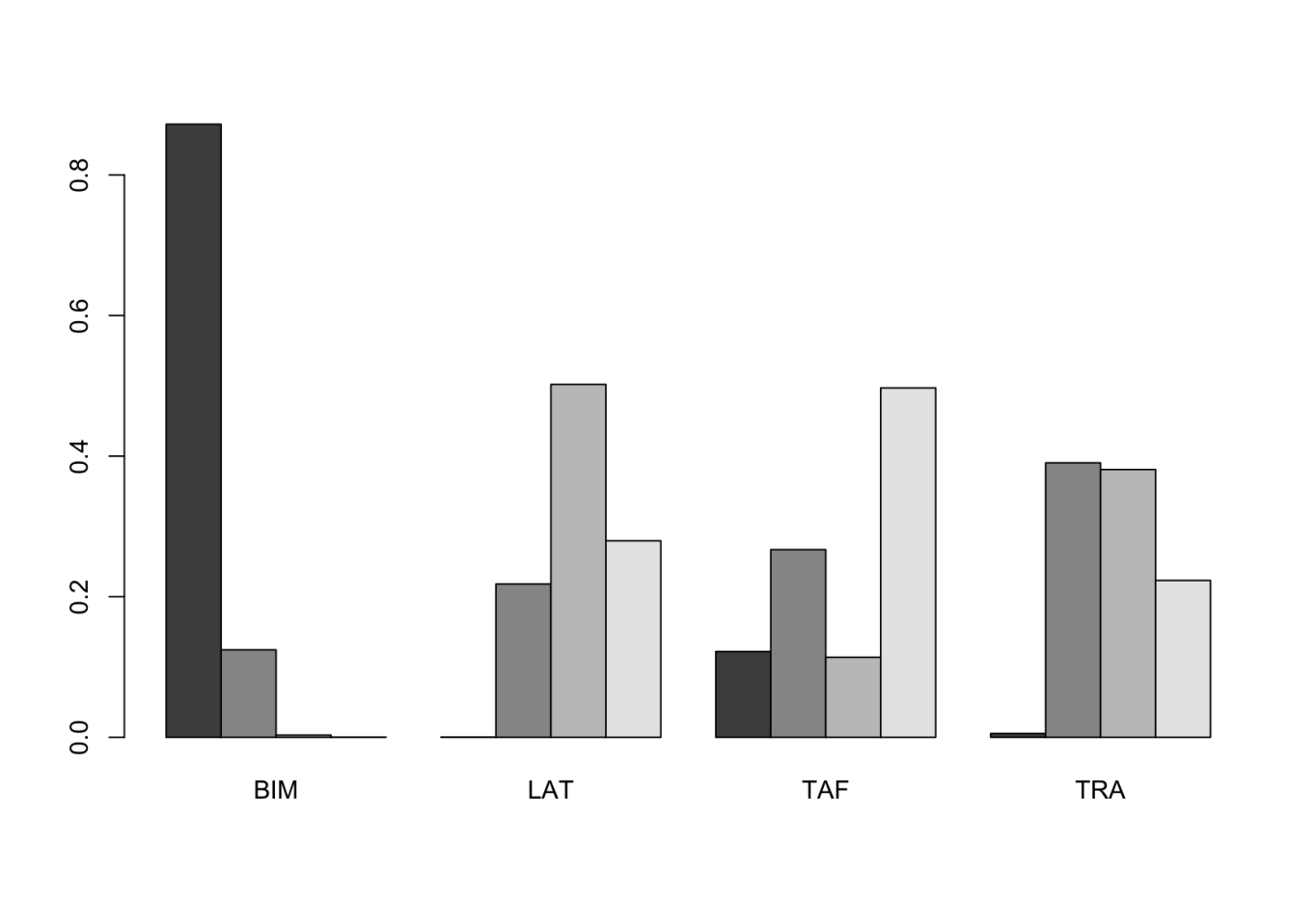
**

| **Prostaglandins** | **BIM** | **LAT** | **TAF** | **TRA** |
| --- | --- | --- | --- | --- |
| **SUCRA** | **95.6%** | **31.3%** | **33.8%** | **39.2%** |

**Figure S9.2**: Cumulative ranking curve plots of Prostaglandins for **Incidence of Conjunctival Hyperemia** in range network. Higher surface under the curve reflects lower probability of association with **Incidence of Conjunctival Hyperemia.**

**
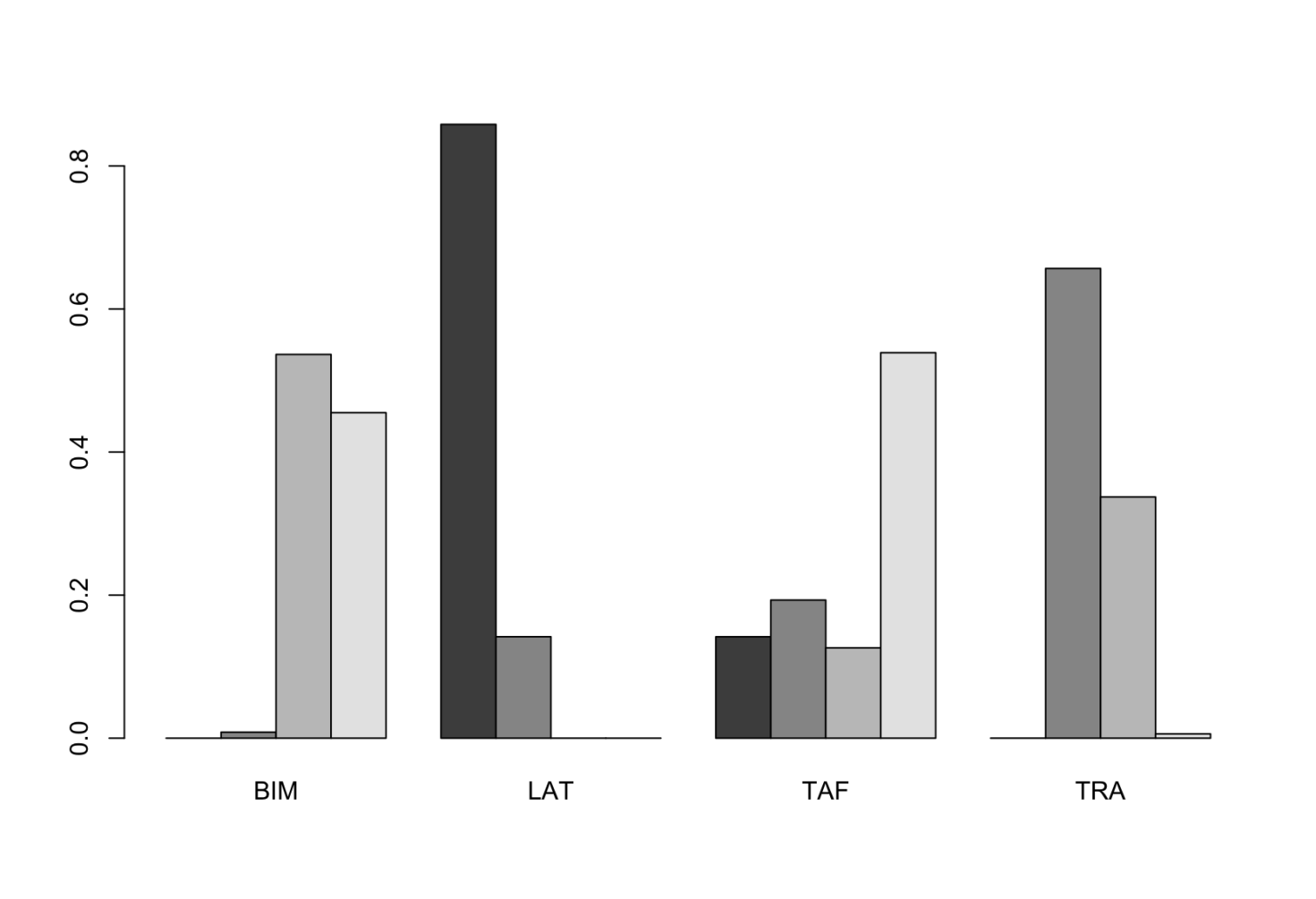
**

| **Prostaglandins** | **BIM** | **LAT** | **TAF** | **TRA** |
| --- | --- | --- | --- | --- |
| **SUCRA** | **18.4%** | **95.2%** | **31.2%** | **55.0%** |

**Appendix 10: League table of Summary Estimates for Prostaglandins on EMs Derived from Network Meta-analysis of 23 Trials**

**Table S10.1: Intraocular Pressure Reduction**

The columns represent the comparison of the row drug class to the column drug class. The rows represent the comparison of the row drug class to the column drug class. The effect estimates are expressed as RR and 95% CI. For example, the MD in **Intraocular Pressure Reduction** for SZD compared to DFP is 2.11(1.29, 3.49). **MD＞0 favors the drug in the column (red), and MD＜0 favors the drug in the row (green).**

| BIM |  |  |  |
| --- | --- | --- | --- |
| **0.69 (0.28, 1.09)** | LAT |  |  |
| 0.74 (-0.56, 1.93) | 0.06 (-1.17, 1.18) | TAF |  |
| **0.64 (0.14, 1.09)** | -0.05 (-0.51, 0.37) | -0.11 (-1.31, 1.17) | TRA |

**Table S10.2: Incidence of Conjunctival Hyperemia**

The columns represent the comparison of the row drug class to the column drug class. The rows represent the comparison of the row drug class to the column drug class. The effect estimates are expressed as RR and 95% CI. For example, the OR in **Incidence of Conjunctival Hyperemia** for SZD compared to DFP is 2.11(1.29, 3.49). **OR＞1 favors the drug in the column (red), and OR＜1 favors the drug in the row (green).**

| BIM |  |  |  |
| --- | --- | --- | --- |
| **3.3 (2.51, 4.38)** | LAT |  |  |
| 0.78 (0.02, 8.01) | 0.24 (0.01, 2.37) | TAF |  |
| **1.51 (1.06, 2.16)** | **0.46 (0.33, 0.63)** | 1.94 (0.18, 68.91) | TRA |

# Appendix 11: Funnel plots

**Figure S11.1: Funnel plot of Intraocular Pressure Reduction**

**
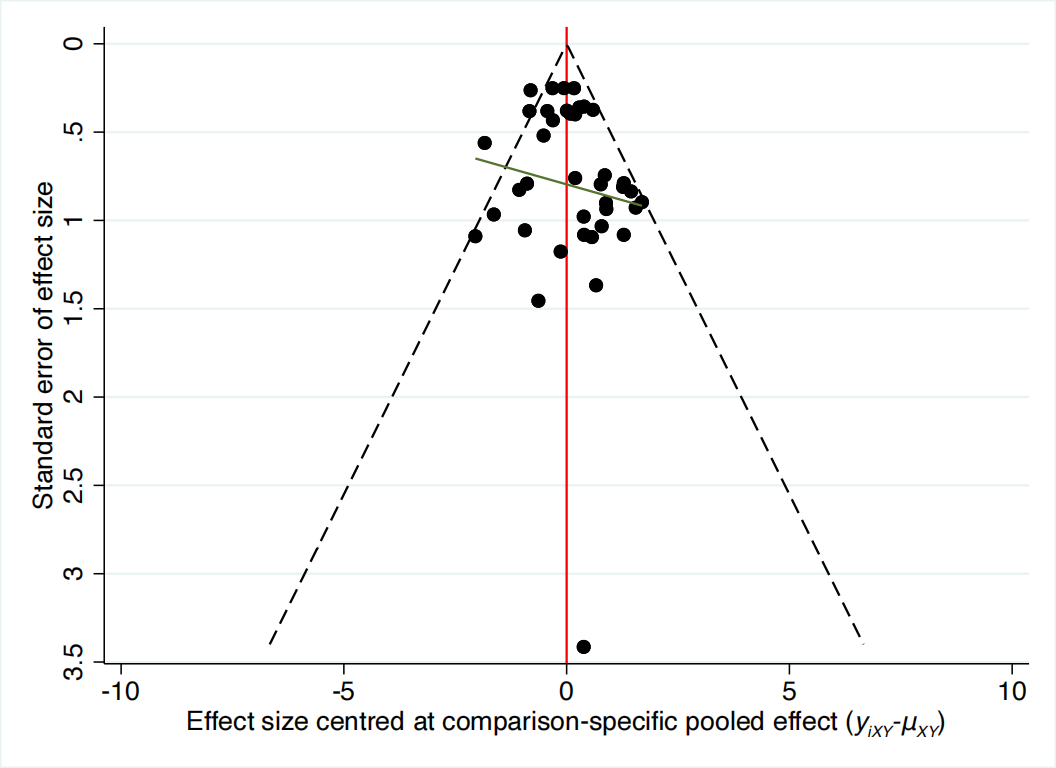
**

**Figure S11.2: Funnel plot of Incidence of Conjunctival Hyperemia**


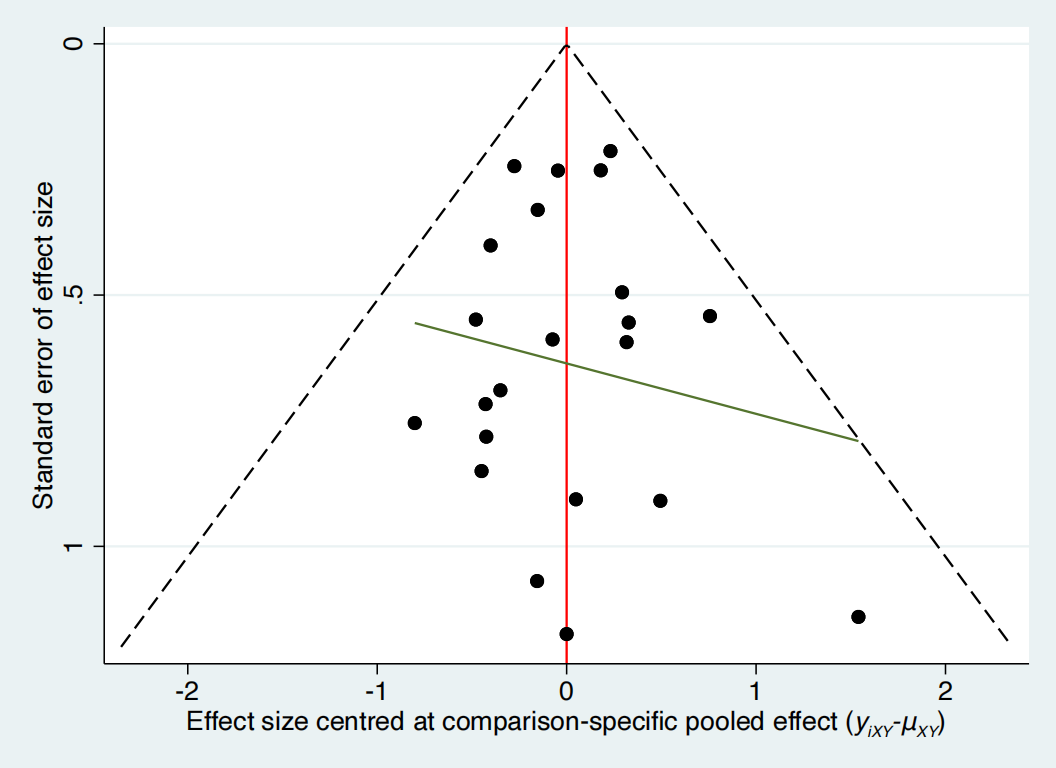


# Appendix 12: Sensitivity analysis

**Table S12.1 Sensitivity Analyses of Intraocular Pressure Reduction**

| _BIM_ | _LAT_ | _TAF_ | _TRA_ |
| --- | --- | --- | --- |
| BIM | -0.71 (-1.07,-0.34) | -0.80 (-1.95,0.34) | -0.68 (-1.12,-0.24) |
| 0.71 (0.34,1.07) | LAT | -0.10 (-1.19,0.99) | 0.02 (-0.39,0.44) |
| 0.80 (-0.34,1.95) | 0.10 (-0.99,1.19) | TAF | 0.12 (-1.03,1.27) |
| 0.68 (0.24,1.12) | -0.02 (-0.44,0.39) | -0.12 (-1.27,1.03) | TRA |

**Table S12.2 Sensitivity Analyses of Incidence of Conjunctival Hyperemia**

| _BIM_ | _LAT_ | _TAF_ | _TRA_ |
| --- | --- | --- | --- |
| BIM | 0.30 (0.23,0.38) | 0.96 (0.09,9.71) | 0.65 (0.48,0.89) |
| 3.35 (2.61,4.31) | LAT | 3.21 (0.32,32.12) | 2.19 (1.67,2.87) |
| 1.04 (0.10,10.57) | 0.31 (0.03,3.11) | TAF | 0.68 (0.07,6.91) |
| 1.53 (1.12,2.10) | 0.46 (0.35,0.60) | 1.47 (0.14,14.93) | TRA |
